# Tissue microenvironment dictates the state of human iPSC-derived endothelial cells of distinct developmental origin in 3D cardiac microtissues

**DOI:** 10.1016/j.isci.2025.113611

**Published:** 2025-09-22

**Authors:** Xu Cao, Maria Mircea, Sara Cascione, Atoosa Amel, Theano Tsikari, Francijna E. van den Hil, Hailiang Mei, Katrin Neumann, Anna Alemany, Konstantinos Anastassiadis, Christine L. Mummery, Stefan Semrau, Valeria V. Orlova

**Affiliations:** 1 Department of Anatomy and Embryology, Leiden University Medical Center, Einthovenweg 20, Leiden 2333ZC, the Netherlands; 2 Leiden Institute of Physics, Leiden University, RA 2333, Leiden, the Netherlands; 3 Sequencing Analysis Support Core, Leiden University Medical Center, Leiden 2333ZA, the Netherlands; 4 Stem Cell Engineering, Biotechnology Center, Center for Molecular and Cellular Bioengineering, Technische Universität Dresden, Dresden 01307, Germany

**Keywords:** Stem cells research, Developmental biology, Transcriptomics

## Abstract

Each tissue and organ in the body has its own type of vasculature. Here, we demonstrate that organotypic vasculature for the heart can be recreated in a three-dimensional cardiac microtissue (MT) model composed of human induced pluripotent stem cell (hiPSC)-derived cardiomyocytes (CMs), cardiac fibroblasts (CFs), and endothelial cells (ECs). ECs in cardiac MTs upregulated expression of markers enriched in human intramyocardial ECs, including *CD36*, *CLDN5*, *APLNR*, *NOTCH4*, *IGFBP3*, and *ARHGAP18*. We further show that the local microenvironment largely dictates the organ-specific identity of hiPSC-derived ECs: we compared ECs derived from cardiac and paraxial mesoderm and found that, regardless of origin, they acquired similar identities upon integration into cardiac MTs. Overall, the results indicated that while the initial gene profile of ECs was dictated by developmental origin, this could be modified by the local tissue environment. This developmental “plasticity” in ECs has implications for multiple pathological and disease states.

## Highlights

• Compared hiPSC-derived endothelial cells (ECs) from cardiac vs. paraxial mesoderm
• ECs isolated on day 6 and day 8 retained their developmental lineage history
• No organ-specific EC signature in day 6 or day 8 cardiac/paraxial mesoderm ECs
• 3D Cardiac microtissues support organ-specific EC signature in cardiac/paraxial ECs

## Graphical abstract

**Figure:**
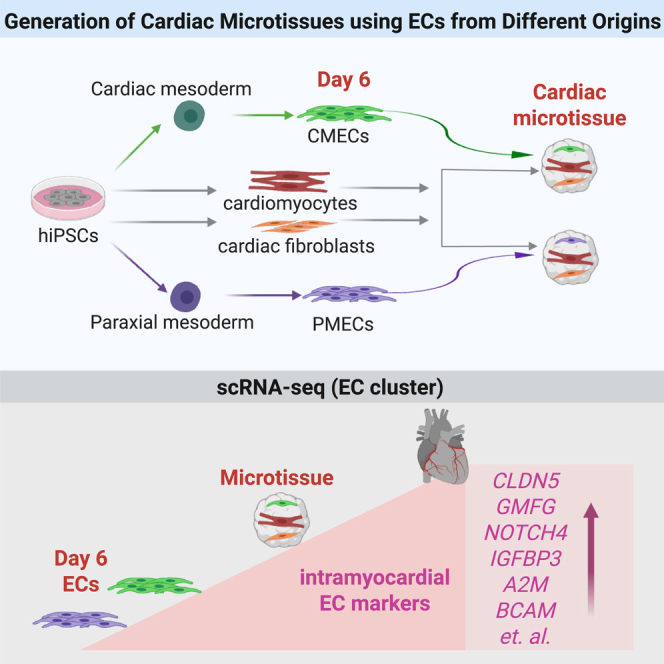

## Introduction

Development of the vascular system is one of the earliest events in organogenesis and defects in this process often result in embryonic and postnatal lethality. Endothelial cells (ECs) that form the inner lining of blood and lymphatic vessels are specialized cells that adapt to local microenvironmental cues to support the function of various organs. The heart is a prime example of the importance that interplay between the vasculature and the myocardium has in organ growth, remodeling, and function. ECs in the heart originate from several developmental lineages that later converge to similar states depending on their location. Structurally and functionally, ECs in the heart can be divided into the endocardium, intramyocardial capillary ECs, coronary arteries/veins and lymphatic ECs. During development, heart ECs predominantly originate in the lateral plate mesoderm that includes both pre-cardiac and cardiac mesoderm.^1^ Endocardium and *sinus venosus* are the two major sources of intramyocardial and coronary ECs.^2^^,^^3^^,^^4^^,^^5^^,^^6^ Furthermore, genetic ablation of the *sinus venosus* EC lineage results in compensation from the endocardial EC lineage.^5^ Endocardium- and *sinus venosus-*derived EC progenitors, converge to an increasingly similar state that is dictated by the local microenvironment, despite being initially transcriptionally distinct.^7^ In addition, recent studies showed that cardiac lymphatic ECs predominately originate from two lineages, namely Isl1+ second heart field and Pax3+ paraxial mesoderm (PM),^8^^,^^9^ which further increases the spectrum of developmental origins of ECs in the heart.

Endothelial cells in the intramyocardial capillaries, or intramyocardial ECs, constitute a specialized barrier between the blood and the myocardium. They deliver oxygen and essential nutrients, specifically fatty acids, to the cardiomyocytes to fulfill high energy demands of the working myocardium.^10^ Endocardial ECs form the lining of the inner surface in the ventricles and atria and play important roles during the development of working myocardium, such as formation of trabeculae network and compaction of the myocardium.^11^ Analysis of developing mouse heart identified markers that distinguish endocardial and intramyocardial ECs.^12^^,^^13^ Recent single-cell RNA sequencing (scRNA-seq) studies of the human fetal heart confirmed that some of the markers identified in mouse are conserved in human, including *CD36* and *FABP5* for intramyocardial ECs and *NPR3* and *CDH11* for endocardial cells, among others.^14^^,^^15^^,^^16^

Human pluripotent stem cells (hPSCs) represent a valuable *in vitro* model to study early stages of human development, including the heart.^17^^,^^18^ Over the past several years, protocols to differentiate cardiac cell types from hPSCs, such as different sub-types of cardiomyocytes, epicardial cells, cardiac fibroblasts, and ECs were developed.^19^ Engineered multicellular cardiac tissues can be generated by combining these different cell types.^20^ These have proved useful to investigate the contribution of non-cardiac cell types to cardiomyocyte cell maturation and disease.^21^ In addition, methods to create multicellular cardioids from hPSCs have been developed to model embryonic stages from self-organized cardiac and foregut structures to early morphogenesis during heart tube formation.^22^^,^^23^^,^^24^^,^^25^^,^^26^ Recent studies showed that by following a specialized developmental program, hPSCs can be differentiated into ECs from several developmental lineages, such as extra- and intraembryonic hemogenic ECs,^27^^,^^28^^,^^29^ endocardial ECs,^30^ and liver sinusoidal ECs.^31^^,^^32^ We previously developed a method to co-differentiate cardiomyocytes and ECs from hPSCs from cardiac mesoderm.^33^ These hPSC-derived ECs expressed a number of cardiac specific genes like *MEOX2*, *GATA4*, *GATA6*, and *ISL1*, while tissue specific endocardial or intramyocardial markers were still absent, likely because of the lack of local microenvironmental cues.

Recently, we established a three-dimensional (3D) cardiac microtissue (MT) model composed of ECs, cardiomyocytes and cardiac fibroblasts, all derived from hiPSCs.^21^ We showed that hPSC-derived cardiomyocytes in cardiac 3D MTs showed enhanced functional and structural maturation via interaction with ECs and cardiac fibroblasts. We further showed that developmental origin of the fibroblasts was critical in this model, as cardiac-, but not skin-, fibroblasts supported cardiomyocyte maturation in 3D MTs.

However, whether 3D cardiac MTs actually induce organ-specific characteristics and to what extent the developmental origin of ECs plays a role has not been investigated. We therefore aimed here to address these questions by comparing ECs derived from two distinct mesoderm sub-types: MESP1+ cardiac mesoderm and PAX3+ paraxial mesoderm. To do this, we utilized our earlier protocol to differentiate cardiac mesoderm-derived ECs^33^ and developed an optimized protocol to differentiate ECs from paraxial mesoderm. Cardiac MTs were then generated using these two sources of ECs. Although newly differentiated ECs from the two origins showed distinct identities, they strikingly became more similar after extended culture in cardiac MTs. Furthermore, based on endocardial and intramyocardial EC-specific signatures extracted from a published scRNA-seq dataset of human fetal heart,^34^ we observed an intramyocardial EC rather than an endocardial EC identity for both developmental origins after MT culture. In summary, this study shows that although certain characteristics are inherited from progenitors, ECs are “plastic” and efficiently adapt to the microenvironment to acquire new tissue-specific signatures. Our results provide deeper insights into how organ/tissue-specific cell identities are acquired; this will inform the preparation of hiPSC -derived, organ specific ECs for disease modeling and drug development but as importantly, will provide a platform for understanding how EC plasticity might be regulated by microenvironmental context.

## Results

### Differentiation of endothelial cells from cardiac and paraxial mesoderm

**Figure 1:**
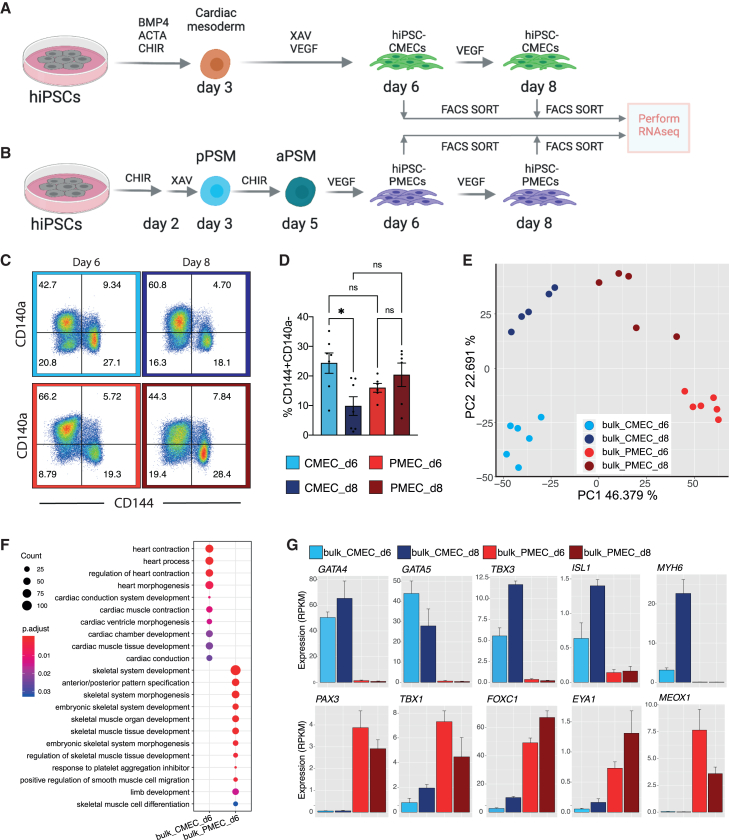
Characterization of ECs differentiated from hiPSCs using CMEC and PMEC protocols (A and B) Schematic overview of CMEC (A) and PMEC (B) differentiation protocols. Endothelial cell (CD144+CD140a-) were FACS sorted on days 6 and day 8 for bulk RNA-seq. ACTA: activin-A. CHIR: CHIR99021. pPSM/aPSM: posterior/anterior presomitic mesoderm. LPM: lateral plate mesoderm. (C) Flow cytometry analysis of CD140a and CD144 expression on day 6 and day 8 of CMEC and PMEC differentiation. (D) Quantification of VEC (CD144)+CD140a-cells on day 6 and day 8 of CMEC and PMEC differentiation. Data are shown as mean ± SD from five to six independent differentiations. (E) PCA analysis of ECs sorted on day 6 and 8 of the CMEC or PMEC protocol. (F) GO term enrichment analysis for DEGs between CMECs and PMECs on day 6 of differentiation. Complete list of GO terms can be found in Table S2. Color represents the p_adjusted_ of enrichment analysis and dot size represents the count of genes mapped to the GO term. (G) Normalized gene expression levels (RPKM) of cardiac and skeletal related genes in CMECs and PMECs on day 6 and 8. Data are shown as mean ± SD from five to six independent differentiations. See also Figure S1, Tables S1 and S2.

We set out to derive ECs from human induced pluripotent stem cells (hiPSCs) via both cardiac and paraxial mesoderm intermediates. To obtain ECs from cardiac mesoderm (CMECs), we used a protocol established previously in our group (Figure 1A).^33^ Briefly, BMP4 (20 ng/mL), activin A (ACTA, 20 ng/mL) and CHIR99021 (CHIR, 1.5 μM) were used to induce cardiac mesoderm from day 0 till day 3. XAV-939 (XAV, 5 μM) and VEGF (50 ng/mL) were used to induce CMECs and early cardiomyocytes (CMs) from day 3 to day 6. For paraxial mesoderm-ECs (PMECs), we adapted a protocol developed by Loh et al. (Loh et al., 2016) (Figure 1B). Briefly, high CHIR (8 μM) was used for the first two days followed by XAV (5 μM) for one day to induce posterior presomitic mesoderm (pPSM) on day 3 and low CHIR (1.5 μM) was used to induce anterior presomitic mesoderm (aPSM) from day 3 to day 5. VEGF was added to induce PMECs from day 5 to day 6. In order to characterize paraxial mesoderm differentiation and the PMEC lineage we established a double fluorescent hiPSC reporter line (NCRM1 PAX3^Venus^MSGN1^mCherry^) (Figure S1A). CRISPR-Cas9 assisted gene editing was used to target the fluorescent protein venus to the PAX3 locus leading to transcriptional control by the endogenous PAX3 regulatory elements. The MSGN1^mCherry^ reporter was generated using a BAC construct integrated into the cells using the piggyBac transposon system. Flow cytometry analysis at different stages of paraxial mesoderm differentiation showed efficient induction of pPSM (MSGN-mCherry positive cells) and aPSM (PAX3-Venus positive cells) on day 2–3 and day 5, respectively (Figure S1B). Using our paraxial mesoderm protocol, more than 70% of cells acquired MSGN1-mCherry expression on day 2–3 (Figure S1C) and more than 50% of cells acquired PAX3-Venus on day 6 (Figure S1D). We next confirmed induction of endogenous PAX3 protein expression by immunostaining with a PAX3-specific antibody (Figure S1E). Notably, more than 90% of cells were positive for PAX3 on day 5; this could be because of relatively weak endogenous expression of Venus that could not be detected in PAX3^low^ cells.

Gene expression analysis further confirmed comparable expression of pan-mesoderm markers *TBXT* and *MIXL1* in both cardiac and paraxial mesoderm differentiation conditions. On the other hand, expression of cardiac genes (*MESP1*, *GATA4*, and *NKX2-5*) was restricted to cardiac mesoderm differentiation conditions and expression of paraxial mesoderm genes (*MSGN1*, *TBX6*, and *PAX3*) was restricted to paraxial mesoderm differentiation conditions (Figure S1F).

Both cardiac and paraxial mesoderm differentiation conditions resulted in comparable percentages of CD144+CD140a- ECs on day 6 and day 8 of differentiation (Figures 1C and 1D). CD144+CD140a- ECs were sorted on day 6 and day 8 of differentiation from both cardiac and paraxial mesoderm conditions and underwent RNA sequencing (RNA-seq). Principal-component analysis (PCA) showed that CMECs and PMECs clustered separately along PC1, and day 6 and day 8 were separated along PC2 (Figure 1E). On day 6, 3307 and 2592 genes were significantly differentially upregulated (FDR<0.05, fold-change>2) in CMECs and PMECs, respectively (Table S1). Gene ontology (GO) enrichment of biological processes showed that cardiac related genes were specifically upregulated in day 6 CMECs (bulk_CMEC_d6), while genes related to skeletal system development and function were specifically upregulated in day 6 PMECs (bulk_PMEC_d6) (Figure 1F, Table S2). Genes involved in heart development, like *GATA4*, *GATA5*, *TBX3*, *ISL1*, and *MYH6*, were highly expressed in day 6 and day 8 CMECs. *TBX3*, *ISL1*, and *MYH6* were upregulated from day 6 to day 8 in CMECs (Figure 1G). Essential genes for skeletal muscle development like *PAX3*, *TBX1*, *FOXC1*, *EYA1*, and *MEOX1* were largely expressed in day 6 and day 8 PMECs. *FOXC1* and *EYA1* were upregulated from day 6 to day 8, while *TBX1* and *MEOX1* were downregulated (Figure 1G). In summary, unbiased expression analysis by bulk RNA-seq showed differential gene expression signatures of cardiac and paraxial mesoderm derived ECs that corresponded to their known expression profiles *in vivo*.

### Reconstruction of the differentiation trajectories of ECs by single-cell RNA-seq

**Figure 2:**
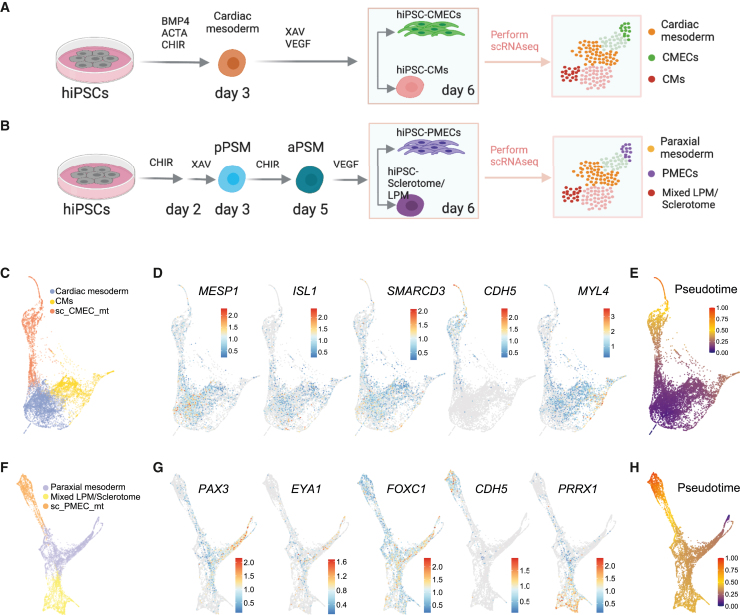
Single-cell RNA sequencing analysis of ECs differentiated from cardiac and paraxial mesoderm (A and B) Schematic overview of CMEC (A) and PMEC (B) differentiation protocols until day 6. Cells were collected for scRNA-seq on day 6. ACTA: activin-A. CHIR: CHIR99021. pPSM/aPSM: posterior/anterior presomitic mesoderm. LPM: lateral plate mesoderm. (C) scRNA-seq of CMEC differentiation on day 6 (PAGA plot). Three clusters of cells, indicated by color, were identified. (D) PAGA plots show expression of *MESP1*, *ISL1*, *SMARCD3*, *CDH5*, and *MYL4* on day 6 of CMEC differentiation. Color represents log transformed expression. (E) Diffusion pseudotime analysis of CMEC differentiation on day 6. (F) scRNA-seq of PMEC differentiation on day 6 (PAGA plot). Three clusters of cells, indicated by color, were identified. (G) PAGA plots show expression *PAX3*, *EYA1*, *FOXC1*, *CDH5*, and *PPRX1* on day 6 of CMEC differentiation. Color represents log transformed expression. (H) Diffusion pseudotime analysis of PMEC differentiation on day 6. See also Figures S2 and S3, Tables S3 and S4.

Having demonstrated that the two differentiation protocols result in ECs with distinct characteristics, we undertook an unbiased analysis of the complete cell population and reconstructed the EC differentiation trajectories. To this end, we performed scRNA-seq on day 6 of CMEC and PMEC differentiation from two independent biological replicates (Figures 2A, 2B, S2A, and S2B). The replicates appeared highly similar in a low-dimensional representation (Figure S2C) and were therefore combined for further analysis. Any remaining, undifferentiated hiPSCs were excluded from further analysis (Figures S2D and S2E). In the CMEC differentiation dataset, cells were grouped into 3 clusters using Louvain clustering with the resolution parameters 0.4 (cardiac mesoderm, cardiomyocytes, and CMECs), as established previously^35^ (Figure 2C). The three cell types were identified by known marker genes (Figures 2D, S3A and S3B, Table S3). The cardiac mesoderm cluster was characterized by mesoderm and early cardiac genes, such as *MESP1*, *SMARCD3*, *ABLIM1*, *TMEM88*, *ISL1*, and *MYL5*, as well as the cell cycle-related genes *CDK6* and *NEK2*. The CMEC cluster was characterized by EC markers (*CDH5*, *CD34*, *KDR*, *HEY2*, *TEK*, *TIE1*, *ACVRL1*, *SOX17*, *ENG*, *ICAM2*, and *PECAM1*). Cardiomyocytes were identified by expression of cardiomyocyte-associated genes, including *MYL4*, *TNNI1*, *MYL7*, *ACTA2*, *TNNT2*, *HAND2*, and *NKX2-5*. To reveal the differentiation trajectories of the cells, we calculated the diffusion pseudotime using a cardiac cell mesoderm cell as root (Figure 2E). Pseudotime increased toward CMECs and cardiomyocytes, suggesting that both cell types differentiated from a common cardiac mesoderm progenitor.

In the PMEC differentiation dataset, all cells were divided into 3 clusters using Louvain clustering with the resolution parameters 0.3 (Figure 2F), which were interpreted as being paraxial mesoderm, PMECs and mixed lateral plate mesoderm (LPM)/sclerotome using marker gene analysis (Figures S3C and S3D, Table S4). The paraxial mesoderm cluster was characterized by expression of aPSM and dermomyotome genes, such as *MEOX1*, *PDGFRB*, *SIX1*, *CRABP2*, *NR2F1*, *EYA1*, *FOXC1* and *PAX3*. PMECs were characterized by EC markers, like *ETV2*, *CDH5*, *CD34*, *KDR*, *ENG*, *SOX17*, *PLVAP*, *APLN*, *NRP1*. The mixed LPM/sclerotome cluster was characterized by LPM and sclerotome specific genes, such as *TMEM88*, *HAND1*, *TNNI1*, *PRRX1*, *ACTA2*, *DES*, *FOXH1*, *LEF1*, and *JAG1* (Figures 2G, S3C and S3D). Diffusion pseudotime rooted in the paraxial mesoderm increased toward both PMECs and LPM/sclerotome (Figure 2H). Both cell types therefore likely differentiated from a common paraxial mesoderm progenitor.

### Acquisition of an organ-specific identity in cardiac microtissues

**Figure 3:**
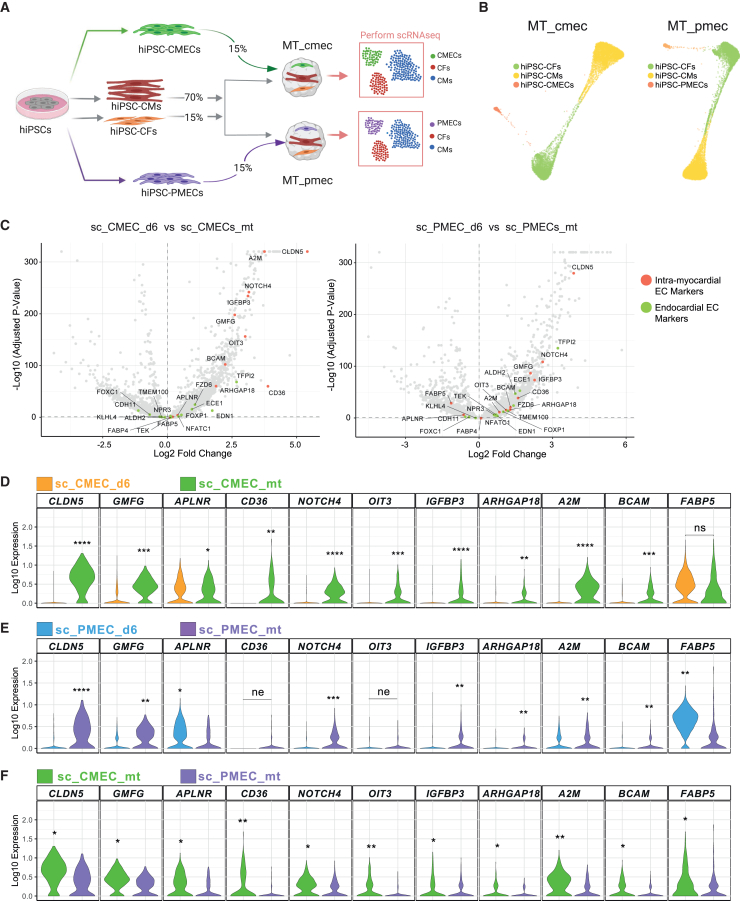
hiPSC-ECs acquired organ-specific signatures in a cardiac microenvironment (A) Generation of cardiac MTs from hiPSC-CMs, hiPSC-CFs, and hiPSC-ECs. CMECs and PMECs were used for MT_cmec and MT_pmec, respectively. MTs were collected after 21 days for scRNAseq. (B) scRNAseq data of MT_cmec (left) and MT_pmec (right) were visualized using PAGA. Three clusters of cells were identified in both datasets. (C) Volcano plot showing fold changes and *p* values of differential expression tests between sc_CMEC_d6 and sc_CMEC_mt (left), or sc_PMEC_d6 and sc_PMEC_mt (right). Representative intra-myocardial and endocardial markers that are differentially expressed (p_adjusted_ < 0.05) are highlighted in red and green, respectively. (D–F) Violin plots of gene expression in sc_PMEC_ d6, sc_CMEC_d6, sc_CMEC_mt, and sc_PMEC_mt for representative Intra-myocardial EC markers. Asterisks indicate significance level of differential gene expression tests between the respective populations. ns: *p* > 0.05; ∗*p* ≤ 0.05; ∗∗*p* ≤ 1e-10; ∗∗∗*p* ≤ 1e-100; ∗∗∗∗*p* ≤ 1e-200. The clusters with higher expression value were labeled. ne: not expressed (0 counts) in >85% of cells in both groups. See also Figures S4 and S5, Tables S5, S6, S7, and S8.

Being able to produce ECs with properties corresponding to their mesodermal origins enabled us to test in how far the cellular microenvironment can either reinforce or reverse this specification i.e., how “plastic” the ECs are. Specifically, we set out to mimic the cardiac microenvironment *in vitro* using a protocol for creating cardiac MTs, published previously by our group.^21^ Briefly, CD34^+^ CMECs or PMECs were sorted on day 6 and combined with hiPSC-derived cardiomyocytes (hiPSC-CMs) and hiPSC-derived fibroblasts (hiPSC-CFs) in a ratio of 15:70:15 to form MTs. MTs made from CMECs (MT_cmec) and PMECs (MT_pmec) were collected after 21 days from two independent biological replicates by scRNA-seq (Figures 3A, S2A and S2B). The replicates appeared highly similar in a low-dimensional representation (Figure S2C) and were therefore combined for further analysis. Any remaining, undifferentiated hiPSCs were excluded from further analysis (Figures S2F and S2G). Both MT_cmec and MT_pmec datasets were divided into three clusters that correspond to hiPSC-CFs, hiPSC-CMs and hiPSC-ECs using Louvain clustering with the resolution parameters 0.4 and 0.1, respectively (Figure 3B). Marker genes identified for each cluster, confirmed the cluster identities (Table S5).

In order to assess to what extent ECs in MTs acquired an organ-specific identity, we compared their expression profiles to primary ECs in a published dataset of the human fetal heart (Figure S4A).^34^ In these dataset, we reannotated the original endothelium/pericytes/adventia cluster (cluster 10) as intramyocardial ECs, based on differentially expressed markers such as *A2M*, *CD36*, *APLNR*, *ARHGAP18*, *IGFBP3*, *CLDN5*, *FABP4*, and *FABP5* (Figure S4B, Table S6). The cluster annotated as capillary endothelium (cluster 0) in the original publication was reannotated as endocardium, due to the presence of differentially expressed markers like *NPR3*, *ALDH2*, *CDH11*, *ECE1*, *TMEM100*, *FOXC1*, and *EDN1* (Figure S4B, Table S6). Supporting the differential expression test, UMAP visualization of representative intra-myocardial and endocardial markers showed specific expression in the respective clusters (Figures S4C and S4D).

We next compared CMECs and PMECs on day 6 of differentiation (sc_CMEC_d6 and sc_PMEC_d6) with the CMECs and PMECs in MTs (sc_CMEC_mt and sc_PMEC_mt) respectively, with a cutoff of *p* value <0.05 (adjusted by the Benjamini-Hochberg method) for the differentially expressed genes (DEGs). CMECs in MTs upregulated expression of intramyocardial makers, such as *CLDN5*, *GMFG*, *APLNR*, *CD36*, *NOTCH4*, *OIT3*, *IGFBP3*, *ARHGAP18*, *A2M*, and *BCAM*, but not *FABP5*, compared to CMECs isolated on day 6 of differentiation (Figures 3C and 3D, Table S7). PMECs in MTs upregulated expression of a few intramyocardial markers, such as *CLDN5*, *GMFG*, *NOTCH4*, *IGFBP3*, *ARHGAP18*, *A2M*, and *BCAM*, but not *APLNR*, *CD36*, *OIT3*, and *FABP5* compared to PMECs isolated on day 6 of differentiation (Figures 3C and 3E, Table S7). We also found some endocardial markers upregulated in both CMECs and PMECs in MTs (*TFPI2*, *EDN1*, *ECE1*, and *FOXP1*) (Figures S5A and S5B, Table S7). However, the differences in endocardial marker expression were smaller compared to intramyocardial markers. Notably, the expression of several intramyocardial markers, especially *APLNR*, *CD36*, *OIT3*, *ARHGAP18*, *A2M*, *BCAM*, and *FABP5* was higher in CMECs in MTs compared to PMECs in MTs (Figure 3F, Table S8). Meanwhile, the expression of endocardial markers was lower in CMECs in MTs compared to PMECs in MTs, although their average expression levels were lower in general when compared to intramyocardial markers (Figure S5C, Table S8). Importantly, endocardial makers, including *CDH11*, *FOXC1*, *FZD6*, *TMEM100*, and *NPR3*, were barely expressed in either CMECs or PMECs in MTs (Figure S5C).

To determine whether the cardiac MT microenvironment is required or whether a prolonged culture alone can lead to the acquisition of heart-specific identity in ECs, CMECs were sorted on day 6 (bulk_CMEC_d6) and day 27 (bulk_CMEC_d27) of differentiation or sorted from MTs on day 27 (bulk_CMEC_mt) after 21 days from three independent biological experiments and analyzed by RNA-seq. These three EC populations exhibited distinct transcriptomic profiles (Figure S6A). A total of 993 genes were upregulated in bulk_CMEC_mt compared to bulk_CMEC_d6, with 166 of them uniquely upregulated in bulk_CMEC_mt but not in bulk_CMEC_d27 (Figure S6B, Tables S9, S10, and S11). GO enrichment analysis showed that genes upregulated in bulk_CMEC_d27 were mainly associated with endothelial development and function, reflecting maturation over time (Table S14). In contrast, bulk_CMEC_mt exhibited strong enrichment for GO terms related to cardiac development and function (Figures S6C and S6D, Table S14). Notably, while intramyocardial EC markers such as *CLDN5* and *FABP5* were upregulated in both bulk_CMEC_d27 and bulk_CMEC_mt, *CD36* was significantly upregulated only in bulk_CMEC_mt (Figure S6E). Overall, both prolonged culture and the cardiac MT microenvironment promoted further maturation of hiPSC-ECs, but the acquisition of an intramyocardial endothelial identity was more pronounced in ECs within the cardiac MTs.

### Distinct cell identities are preserved in cardiac microtissues composed of either cardiac or paraxial mesoderm-derived ECs

**Figure 4:**
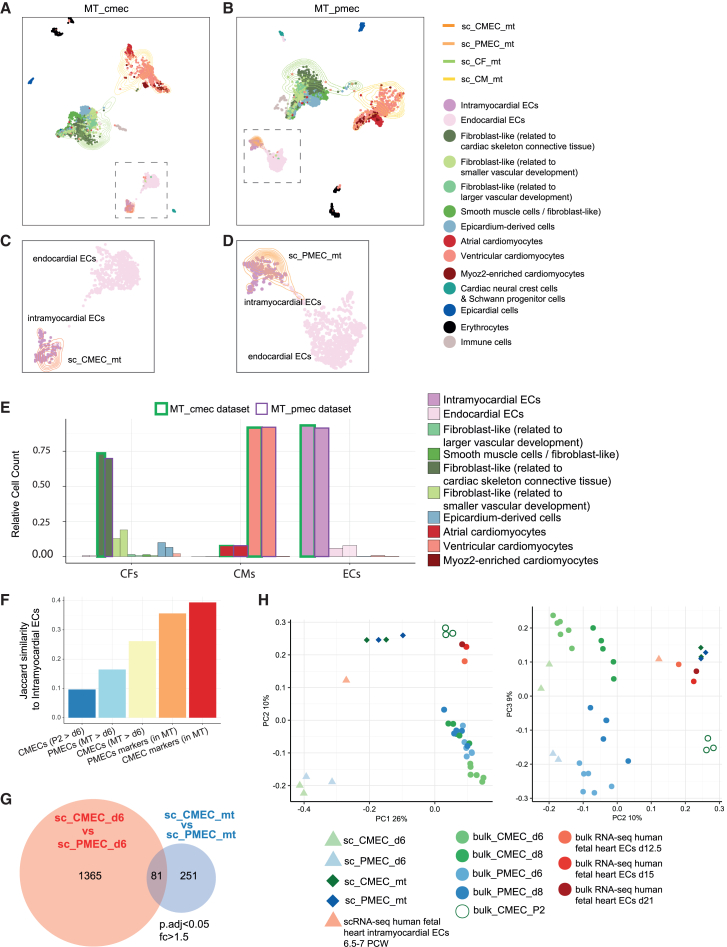
CMECs and PMECs acquired intramyocardial identity in cardiac microenvironment (A and B) Low-dimensional representation (UMAP) of the human fetal heart dataset^34^ integrated with the MT_cmec (A and C) or MT_pmec (B and D) dataset. EC clusters are marked with squares. Cell clusters are indicated by color. Cells in fetal heart dataset are presented with dots and cells in MTs datasets are presented with contour lines. (C and D) Zoom-ins of the EC clusters marked in (A and B). (E) Annotation of CM_MT and PM_MT cells based on nearest neighbors in the *in vivo* dataset. Cells from MT_cmec and MT_pmec are outlined with a green or violet contour, respectively. (F) Jaccard similarity to intramyocardial ECs from the human fetal heart dataset was calculated for each indicated group of genes. CMEC markers (in MT): specific markers of cluster CMECs within the MT_cmec dataset; PMEC markers (in MT): specific markers of cluster PMECs within the MT_pmec dataset; CMECs (MT > d6): DEGs that are higher in sc_CMEC_mt compared to sc_CMEC_d6; PMECs (MT > d6): DEGs that are higher in sc_PMEC_mt compared to sc_PMEC_d6; CMECs (P2 > d6): DEGs that are higher in passage two CMECs compared to sc_CMEC_d6. (G) Venn diagram shows numbers and overlap of DEGs (p_adjusted_ < 0.05 and fold-change >1.5) between CMECs and PMECs from day 6 (in red) and MTs (in blue). (H) PCA plot of different EC populations in scRNA-seq (triangle and diamond) and bulk RNA-seq (circle) datasets. Average expression values of all cells in the cluster were used for the scRNA-seq data. See also Figure S6, Tables S9, S10, S11, S12, and S13.

To obtain a clearer view of the similarities between ECs in MTs and primary fetal heart ECs, we merged the MT_cmec and MT_pmec datasets with the human fetal heart dataset (Figures 4A and 4B).^34^ In the case of both the MT_cmec and the MT_pmec dataset, we found that CFs in MTs (sc_CF_mt) clustered together with fetal heart fibroblast-like cells and CMs in MTs (sc_CM_mt) clustered together with fetal heart ventricular CMs. Notably, both CMECs in MTs (sc_CMEC_mt) and PMECs in MTs (sc_PMEC_mt) clustered together with fetal heart intramyocardial ECs and not endocardium (Figures 4A–4D). To quantify our observation, we calculated the distances (in expression space) between each cell in MTs and the fetal heart dataset. This calculation showed that sc_CF_mt cells are closest to fibroblast-like cells *in vivo* (related to cardiac skeleton connective tissue), sc_CM_mt cells are closest to ventricular cardiomyocytes and sc_CMEC_mt as well as sc_PMEC_mt are closest to intramyocardial ECs in human fetal heart (Figure 4E). Annotating the *in vitro* cells based on the closest *in vivo* neighbors revealed that cell type identities were very similar in MT_cmec and MT_pmec (Figure 4E).

Correspondingly, the set of markers of the EC cluster in MT_cmec and the MT_pmec, sc_CMEC_mt and sc_PMEC_mt, respectively, showed a high overlap using the Jaccard similarity analysis with the markers of intramyocardial ECs we extracted from the *in vivo* dataset (Figure 4F). The Jaccard similarity shows the ratio between common genes to all genes between two sets. The gene set upregulated in sc_CMEC_mt compared to sc_CMEC_d6 had a higher overlap with intramyocardial EC markers than the set of genes upregulated in sc_PMEC_mt compared to sc_PMEC_d6 (Figure 4F). For comparison, we also profiled CMECs that were cultured for two additional passages in monoculture. Genes that were upregulated in these cells compared to sc_CMEC_d6 overlapped the least with intramyocardial EC markers (Figure 4F). This result excludes the possibility that the effects observed in MTs are simply due to environment-independent differentiation progression over time. sc_CMEC_mt and sc_PMEC_mt thus both resembled intramyocardial ECs but a difference between the two differentiation methods remained. To quantify this difference directly, we used differential gene expression analysis. On day 6 of differentiation, 1446 genes were differentially expressed between CMECs and PMECs, while only 332 genes were differentially expressed between sc_CMEC_mt and sc-PMEC_mt (Tables S8, S12, and S13). 81 genes were shared between the two sets and 251 genes were differentially expressed only between sc_CMEC_mt and sc_PMEC_mt (Figure 4G). Intramyocardial marker genes (*CD36*, *OIT3*, *A2M*, *CLDN5*, *APLNR*, and *FABP5*) were among these 251 genes that were differentially expressed between the sc_CMEC_mt and sc_PMEC_mt. This is also in line with our observations that expression of some intramyocardial marker genes were higher in CMECs in MTs compared to PMECs in MTs. Next, all EC clusters from bulk and single cell RNA-seq datasets were combined and visualized using PCA (Figure 4H). CMECs and PMECs on day 6 clustered far apart, while sc_CMEC_mt and sc_PMEC_mt clustered closely together. Bulk and single cell RNA-seq samples clustered together for both CMECs and PMECs. sc_CMEC_mt and sc_PMEC_mt were found closer to fetal heart intramyocardial ECs and fetal heart ECs (Figure 4H) sequenced in our previous study.^21^ Altogether, analysis of the *in vitro* and *in vivo* datasets demonstrated that the cardiac tissue microenvironment resulted in hiPSC-ECs acquiring an intramyocardial EC identity independent of their developmental origin and, further, gene expression differences due to distinct mesodermal origins were partially removed.

## Discussion

In the present study we derived ECs from hiPSCs from two mesoderm lineages namely LPM and PM, as previous lineage tracing studies have shown that LPM and PM serve as a major source of ECs in the developing embryo.^36^^,^^37^^,^^38^ We showed that ECs isolated on day 6 and day 8 of differentiation retained their developmental lineage history. Transcription factors involved in the heart and skeletal muscle development were highly expressed in CMECs and PMECs, respectively. Additional approaches, including scRNA-seq, showed lineage diversification from a common cardiac and paraxial mesoderm progenitor during differentiation of CMECs and PMECs, respectively. At the same time, an organ-specific EC signature was absent in ECs differentiated either from cardiac or paraxial mesoderm on day 6 and day 8 of differentiation, or upon extended culture.

Local microenvironmental cues result in acquisition of organ-specific characteristics in ECs.^39^ To model the influence of cell-extrinsic factors, we took advantage of our cardiac MT model which mimics the heart-specific microenvironment, as it integrates CMs, CFs and ECs.^21^ Although both CMECs and PMECs acquired an intramyocardial EC identity at transcriptomic level after incorporation into MTs, several intramyocardial EC markers *(APLNR*, *CD36*, *OIT3*, *ARHGAP18*, *A2M*, *BCAM*, and *FABP5*) were more strongly upregulated in CMECs compared to PMECs in cardiac MTs. This may be attributed to the cardiac mesoderm developmental origin of CMECs. A recent study in mouse embryos showed that transcriptional heterogeneity in the sinus venosus (SV) and endocardium-derived intramyocardial ECs declines over time.^7^ Therefore, it would be interesting to investigate whether PMECs require longer culture in cardiac MTs to acquire comparable expression of intramyocardial EC markers as in CMECs. On the other hand, endocardial markers were not detected in either CMECs or PMECs in cardiac MTs. This is in line with previous evidence that the cardiac MTs microenvironment recapitulates myocardial and not endocardial layers of the heart. Importantly, while prolonged culture alone led to the upregulation of some intramyocardial EC markers, it was insufficient for acquisition of a heart-specific endothelial identity, underscoring the potential role of the cardiac microenvironment during the cardiac EC development. Besides, CFs and CMs in both MT_cmec and MT_pmec datasets were highly similar and clustered together with fetal heart fibroblast-like cells and fetal heart ventricular CMs, respectively. This showed that developmental origin of ECs does not influence CF or CM identities in cardiac MTs.

Among EC lineages, PM serves a source of lymphatic ECs in the heart, skin, and lymph node.^9^^,^^40^^,^^41^^,^^42^ PMECs showed increased expression of genes important for the development of lymphatic vasculature, such as *TBX1*, *FOXC1*, *LYVE1*, and *VEGFC.* At the same time*,* expression of the master regulator of lymphatic EC differentiation (*PROX1*) was not detected in PMECs either at day 6 or in cardiac MTs. It would be interesting to explore whether addition of known lymphatic EC growth factors promotes *PROX1* expression in PMECs.

Genetic lineage tracing in mice has identified a variety of developmental origins for organ-specific ECs in different tissues. However, whether developmental origin is a prerequisite- or simply a default developmental route remains an open question. On the other hand, the present and previous studies demonstrate that local microenvironmental cues might play a bigger role in not only the acquisition but also the maintenance of the organ-specific characteristics.^43^ This is also in line with recent study on sinusoidal ECs of the liver.^44^ Earlier studies have demonstrated the importance of the *Gata4* transcription factor in the differentiation of liver ECs and the acquisition of sinusoidal-like identity.^45^^,^^46^ However, scRNA-seq analysis of liver ECs showed that *Gata4* is not restricted to sinusoidal ECs but it is expressed by all ECs in the liver.^44^ Instead, the *c-Maf* transcription factor was restricted to sinusoidal liver ECs and was regulated by BMP9 that is highly expressed by hepatic stellate cells (HSC).^47^ Furthermore, overexpression of c-Maf was sufficient to induce sinusoidal-like characteristics in human umbilical vein ECs (HUVECs). The same is true for heart ECs that are derived from multiple developmental pools of EC progenitors that converge to a similar state over time.^1^^,^^5^^,^^7^

In summary, we demonstrated that ECs, derived from two distinct mesoderm lineages namely LPM and PM, acquire organ-specific characteristics at transcriptomic level upon incorporation into the cardiac MT environment which includes CMs and CFs. We expect that our findings will guide the derivation of organ-specific ECs from hiPSCs in the future and lay the foundation for various biomedical applications, from creating of disease models to transplantation therapies.

### Limitations of the study

Several limitations of our study should be noted. First, the ECs within our MT model likely remain immature and do not fully replicate the properties of intramyocardial ECs found in the human heart. Further refinement of the MT model is therefore necessary, including the incorporation of additional cardiac cell types such as macrophages and smooth muscle cells. Moreover, although ECs can form lumens within MTs,^21^ the absence of flow and shear stress remains a significant limitation that should be addressed in future model iterations.^48^ Additionally, since the organ-specific identities of hiPSC-ECs are primarily inferred from transcriptomic analyses, further validation at the protein level and through functional assays will be necessary in future studies. Finally, further mechanistic studies are needed to clarify how the cardiac MT microenvironment promotes the acquisition of organ-specific identity in hiPSC-ECs, and whether direct cell-cell interactions or paracrine signaling play a role.

## Resource availability

### Lead contact

Further information and requests for resources and reagents should be directed to and will be fulfilled by the lead contact, Dr. Valeria V. Orlova (v.orlova@lumc.nl).

### Materials availability

hiPSC lines are available upon MTA.

### Data and code availability

• The bulk and single cell RNA sequencing datasets have been deposited in the Gene Expression Omnibus with accession number GEO: GSE151427.
• This paper does not report original code.
• Software used to analyze the data are either freely or commercially available. Any additional information associated with the data presented in this paper is available from the lead contact upon request.

## Acknowledgments

This project received funding from the 10.13039/501100000781European Research Council (ERCAdG 323182 STEMCARDIOVASC); European Community’s Seventh Framework Programme (FP7/2007–2013) under grant agreement no. 602423; European Union’s Horizon 2020 Research and Innovation Program under grant agreement no. 668724; Netherlands Organ-on-Chip Initiative, an NWO Gravitation project funded by the Ministry of Education, Culture and Science of the government of the Netherlands (024.003.001); the LymphChip project with project number NWA-ORC 2019 1292.19.019 of the NWA research program “Research on Routes by Consortia (ORC)”, which is funded by the Netherlands Organization for Scientific Research (NWO); the Novo Nordisk Foundation Center for Stem Cell Medicine is supported by Novo Nordisk Foundation grants (NNF21CC0073729). We thank Elisa Giacomelli and Milena Bellin for generation of cardiac microtissues; S.L. Kloet and E.de Meijer (Leiden Genome Technology Center) for help with 10× Genomics experiments (cell encapsulation, library preparation, single-cell sequencing, primary data mapping, and quality control); the LUMC Flow Cytometry Core Facility, and the LUMC Light and Electron Microscopy Facility. Illustrations were created with BioRender.

## Author contributions

X.C., M.M., S.S., and V.V.O. designed the research, analyzed and interpreted results, wrote the manuscript; X.C., S.C., A.Amel, T.T., F.E.v.d.H., A.Alemany, and V.V.O. performed experiments; M.M. and S.S. performed scRNA-seq analysis; K.N. generated MSGN1^mCherry^ hiPSC reporter line; K.A. generated gene targeting constructs for reporter hiPSC lines; X.C. and H.M. performed bulk RNA-seq analysis; C.L.M. designed the research and edited the manuscript.

## Declaration of interests

C.L.M. is co-founder of Ncardia bv.

## STAR★Methods

### Key resources table

**Table:** 

REAGENT or RESOURCE	SOURCE	IDENTIFIER
**Antibodiess**		

Anti-human PAX3 primary antibody	Developmental Studies Hybridoma Bank	Cat#Pax3-c; RRID:AB_528426
Donkey anti-Mouse IgG (H + L) Highly Cross-Adsorbed Secondary Antibody, Alexa Fluor™ 488	Thermo Fisher Scientific	Cat#A-21202; RRID:AB_141607
Anti–human VE-cadherin–Alexa Fluor 488	eBioscience	Cat#53–1449; RRID:AB_10753926
Anti–human PDGFR-β-phycoerythrin (PE)	BD Biosciences	Cat#558821; RRID:AB_397132
anti-human CD31 Antibody-FITC	Miltenyi Biotec	Cat#130-117-390; RRID:AB_2733637

**Chemicals, peptides, and recombinant proteins**		

TeSR™-E8™ meidium	Stem Cell Technologies	Cat#05990
Matrigel hESC-Qualified Matrix	Corning	Cat#354277
Vitronectin, truncated recombinant human (VTN-N)	Thermo Fisher Scientific	Cat#A14700
Fibronectin bovine plasma	Sigma Aldrich	Cat#F1141
RevitaCell Supplement (100×)	Thermo Fisher Scientific	Cat#A2644501
UltraPure 0.5 M EDTA, pH 8.0	Thermo Fisher Scientific	Cat#15575020
Recombinant Human BMP-4 Protein	R&D Systems	Cat#314-BP
Human Activin A, premium grade	Miltenyi Biotec	Cat#130-115-010
CHIR99021	Axon Medchem	Cat#Axon1386
XAV939	Tocris	Cat#3748/10
Human VEGF, premium grade	Miltenyi Biotec	Cat#130-109-386
Retinoic Acid	Sigma Aldrich	Cat#R2625
TGFb inhibitor, SB431542	Tocris	Cat#1614/10
Human FGF-2, premium grade	Miltenyi Biotec	Cat#130-093-842
Fibroblast Growth Medium 3	PromoCell	Cat#C-23025
CryoStor CS10 medium	Stem Cell Technologies	Cat#07930
Puromycin dihydrochloride	Sigma Aldrich	Cat#P7255
Collagenase type II	Worthington	Cat#LS004176

**Critical commercial assays**		

EasySep Human CD34 Positive Selection Kit II	Stem Cell Technologies	Cat#17856
Lipofectamine™ 2000 Transfection Reagent	Thermo Fisher Scientific	Cat#11668019
CD34 MicroBead Kit UltraPure, human	Miltenyi Biotec	Cat#130-100-453
NucleoSpin RNA Kit	Macherey- Nagel	Cat#740955
iScript-cDNA Synthesis kit	Bio-Rad	Cat#170-8889
iTaq Universal SYBR Green Supermix	Bio-Rad	Cat#1725124
EasySep™ Human Cord Blood CD34 Positive Selection Kit II	Stem Cell Technologies	Cat#17896

**Deposited data**		

Gene expression (bulk and single cell RNA-sequencing)	This paper	GEO: GSE151427
Gene expression (single-cell RNA-sequencing) of 6.5 PCW human embryonic cardiac tissue	Asp et al.^34^	European Genome-phenome Archive (EGA): EGAS00001003996
Gene expression (bulk RNA-sequencing) of passage two CMECs (CMECs P2) and human fetal heart ECs at gestation age Week(W)12.5, W15 and W21	Giacomelli et al.^21^	GEO: GSE116464

**Experimental models: Cell lines**		

CTRL1 hiPSC line	LUMC hiPSC core facility	LUMC0020iCTRL-06 https://hpscreg.eu/cell-line/LUMCi028-A Zhang et al.^49^
CTRL hiPSC line NCRM1	NIH Center for Regenerative Medicine NIHCRM, obtained from RUDCR Infinite Biologics at Rutgers University	Guadix et al.^50^

**Oligonucleotides**		

Primer sequence for RT-qPCR and VASAseq are shown in Tables S15 and S16	–	–

**Recombinant DNA**		

pCas9-GFP	Addgene	Cat#44719

**Software and algorithms**		

Fiji-ImageJ	PMID: 22743772	https://ImageJ.net/Fiji/Downloads
GraphPad Prism 10	GraphPad	–
RStudio	RStudio	https://rstudio.com/products/rstudio
LUMC BIOPET Gentrap	LUMC Sequencing Analysis Support Core	https://github.com/biopet/biopet
Python	Python Software Foundation	https://www.python.org

### Experimental model and study participant details

#### Cell lines

hiPSC lines LUMC0020iCTRL-06 (female) was generated from primary skin fibroblasts using Sendai virus by the LUMC hiPSC core facility. hiPSC line NCRM1 (male, NIH Center for Regenerative Medicine NIH CRM) was obtained from RUDCR Infinite Biologics at Rutgers University. All hiPSCs were maintained in a humidified 5% CO_2_ incubator, at 37°C. hiPSCs were cultured in TeSR-E8 on Vitronectin XF and were routinely passaged once a week using Gentle Cell Dissociation Reagent (all from Stem Cell Technologies, Vancouver, Canada).

### Method details

#### Generation of PAX3^Venus^MSGN1^mCherry^ reporter line

Prior to targeting, NCRM1 hiPSCs were passaged as a bulk on feeders in hESC- medium.^51^ RevitaCell (Life Technologies, Carlsbad, CA, USA) was added to the medium (1:200) after every passage to enhance viability after single cell passaging with TrypLE (Life technologies). PAX3^Venus^ was generated by CRISPR/Cas9 as follow: NCRM1 hiPSCs were passaged with ratio 1:3 into 60 mm dishes to reach 60–70% confluency the next day for transfection. Cells were transfected with pCas9-GFP (Addgene plasmid #44719), pBR322-U6-hPAX3-gRNA-S1 containing sgRNA CCGGCCAGCGTGGTCATCCT and repair template p15A-cm-hPAX3-Venus-neo-1kb containing a Venus-neo cassette with 1 kb hPAX3 homology arms. The antibiotic selection marker is flanked by FRT sites for Flp-mediated excision. 20 μL lipofectamine (Invitrogen, Waltham, Massachusetts, USA), 8 μg of pCas9-GFP, 8 μg of sgRNA plasmid and 8 μg of linearized repair template were diluted in 600 μL of Opti-MEM and added to each 60 mm dish. After 18 h the medium was changed to hESC medium. After another 6 h G-418 (50 μg/mL) selection was started and was kept for 1 week. Surviving cells were cultured in hESC medium, passaged and transferred into 6-well plates for the transfection of Flp recombinase expression vector to remove the neomycin cassette. 300 μL of Opti-MEM containing 10 μL lipofectamine and 4 μg CAGGs-Flpo-IRES-puro plasmid was added per well for 18 h. Puromycin (0.5 μg/mL) selection was started 24 h post transfection and lasted for 2 days. Once recovered, cells were passage into 96-well format for clonal expansion via limited dilution. Targeted clones were identified by PCR and Sanger sequencing (BaseClear, Leiden, Netherlands). The MSGN1^mCherry^ reporter line was generated by Transposon mediated BAC transgenesis using protocols as previously described.^52^ In brief, a human BAC (RP11-12L16) with piggyBac transposon repeats flanking the bacterial backbone and with mCherry inserted directly after the initiating Methionine of MSGN1 was transfected together with a piggyBac Transposase into NCRM1 hiPSCs.

#### Differentiation of ECs from cardiac and paraxial mesoderm

ECs from cardiac mesoderm were differentiated as previously described.^33^ ECs from paraxial mesoderm were differentiated using a modified Loh et al. protocol.^53^ Briefly, 5 × 10^4^ cells per cm^2^ were seeded on plates coated with 75 μg/mL Matrigel (growth factor reduced) (Corning) the day before differentiation (day −1). At day 0, paraxial mesoderm was induced by changing TeSR-E8 to BPEL (Bovine Serum Albumin [BSA], Polyvinyl alcohol, Essential Lipids) medium,^54^ supplemented with 8 μM CHIR99021. At day 2, cells were refreshed with BPEL supplemented with 5 μM XAV939. At day 3, cells were refreshed with BPEL supplemented with 4 μM CHIR99021. From day 5 onwards, cells were refreshed every 3 days with BPEL medium supplemented with 50 ng/mL VEGF.

#### Differentiation of CMs from hiPSCs

Cardiomyocyte (CM) differentiation was performed in monolayer culture as previously described.^55^ Briefly, hiPSCs were seeded at a density of 25 × 10^3^ cells/cm^2^ onto plates coated with 75 μg/mL growth factor-reduced Matrigel (Corning) one day prior to differentiation (day −1). On day 0, cardiac mesoderm induction was initiated by replacing E8 medium with BPEL medium supplemented with 20 ng/mL BMP4 (R&D Systems), 20 ng/mL ACTIVIN A (Miltenyi Biotec), and 1.5 μM CHIR99021 (Axon Medchem). From day 3 to day 6, cells were cultured in BPEL medium containing 5 μM XAV939 (Tocris), a Wnt signaling inhibitor. Beginning on day 6, the BPEL medium was refreshed every three days. hiPSC-CMs harvested between days 14 and 21, exhibiting >80% purity as determined by flow cytometry for cardiac troponin expression, were used for microtissue fabrication.

#### Differentiation of cardiac fibroblasts (CFs) from hiPSCs

hiPSCs were sequentially differentiated into epicardial cells (EPI) and subsequently into cardiac fibroblasts (CFs), following a previously established protocol.^50^ Briefly, on day −1, cells were seeded at a density of 25 × 10^3^ cells/cm^2^ on Matrigel-coated plates. On day 0, cardiac mesoderm induction was initiated by replacing E8 medium with BPEL medium supplemented with 20 ng/mL BMP4 (R&D Systems), 20 ng/mL ACTIVIN A (Miltenyi Biotec), and 1.5 μM CHIR99021 (Axon Medchem). After three days (day 3), 5 μM XAV939 was added to BPEL medium, along with 30 ng/mL BMP4 and 1 μM retinoic acid (RA; Sigma Aldrich), and this treatment was continued for three days. On day 6, the medium was refreshed with BPEL containing BMP4 (30 ng/mL) and RA (1 μM). On day 9, cells were reseeded at 15 × 10^3^ cells/cm^2^ onto fibronectin-coated plates (2–5 μg/mL) in BPEL medium supplemented with 10 μM SB431542 (Tocris). By day 12, EPI cells were seeded at a density of 25 × 10^3^ cells/cm^2^ on vitronectin-coated tissue culture plates in BPEL medium containing 10 ng/mL FGF2 (R&D Systems). From day 13 onward, the medium was refreshed every two days with BPEL + FGF2 (10 ng/mL). By day 19, cells were transitioned to Fibroblast Growth Medium 3 (FGM3; PromoCell) to promote CF expansion. Medium was refreshed every two days over approximately 10 days. By day 29, the cultures reached confluence with >80% purity (as indicated by COL1A1 immunostaining), and the CFs were ready for use in microtissue fabrication.

#### Generation of 3D cardiac microtissues (MTs)

Cardiac MTs were generated from ECs, CFs and CMs derived from LUMC0020iCTRL06 iPSC line as previously described.^21^ Briefly, on day 6 of CMEC and PMEC differentiation, CD34^+^ ECs were isolated using a Human cord blood CD34 Positive selection kit II (StemCell Technologies) following the manufacturer’s instructions. On the day of MT formation, freshly isolated hiPSC-ECs and cultured hiPSCs-CFs and hiPSC-CMs were combined together to 5000 cells (70% cardiomyocytes, 15% endothelial cells and 15% cardiac fibroblasts) per 50 μL BPEL medium supplemented with VEGF (50 ng/mL) and FGF2 (5 ng/mL). Cell suspensions were seeded on V-bottom 96 well microplates (Greiner bio-one, Kremsmünster, Austria) and centrifuged for 10 min at 1100 rpm. MTs were incubated at 37^o^C, 5% CO_2_ for 21 days with media refreshed every 3–4 days scRNAseq analysis of MTs was performed after 21 days.

#### Prolonged *in vitro* culture of CMECs

In order to compare MT_CMEC with aged-matched CMECs, CD34^+^ cells were isolated on day 6 of CMEC differentiation, using the CD34 MicroBead Kit UltraPure (Miltenyi). The isolated ECs were then cultured in B(P)EL medium supplemented with VEGF (50 ng/mL) for 21 days and FAC-sorted on day 27 for the comparative analysis.

#### Immunofluorescence staining

Cultured cells were fixed in 4% paraformaldehyde for 15 min, permeabilized for 10 min with PBS containing 0.1% Triton X-100 (Sigma-Aldrich) and blocked for 1h with PBS containing 5% BSA (Sigma-Aldrich). Then cells were stained with primary antibody anti-PAX3 overnight at 4°C. The next day, cells were washed three times (20 min each time) with PBS. The cells were then incubated with fluorochrome-conjugated secondary antibodies for 1h at room temperature and washed three times (20 min each time) with PBS. Both primary and secondary antibodies were diluted in 5% BSA/PBS.

#### Fluorescence-activated cell sorting

For FACS sorting on day 6 and day 8 of the CMEC and PMEC differentiation, cells were detached using TrypLE for 5 min at 37°C and washed once with FACS buffer (PBS containing 0.5% BSA and 2 mM EDTA). Primary antibodies CD144 (1:50, eBioscience), CD140a (1:20, BD Bioscience) were added for 1 h at 4^o^C. Samples were measured on MACSQuant VYB (Miltenyi Biotech) equipped with a violet (405 nm), blue (488 nm) and yellow (561 nm) laser. The results were analyzed using Flowjo v10 (FlowJo, LLC). CD144+CD140a-cells were sorted using FACSAria III (BD-Biosciences). On day 27 of differentiation, CMECs were FAC-sorted for CD144+ cells using FACSAria III (BD-Biosciences).

For FACS sorting of CMECs from cardiac MTs, MTs were collected in a 15 mL conical tube and washed with 0.5 mL HBSS (Thermo Fischer Scientific) before being transferred in a 1.5 mL tube. The cardiac MTs were then incubated with 300 μl of collagenase type II solution (290 U/mg in HBSS, pre-warmed to 37°C, Worthington) for 20 min at 37°C with constant shaking at 450 rpm in a heating block. After incubation, the cardiac MTs were gently pipetted three times up and down with a P-1000 pipette and the supernatant containing single cells was transferred to a 15 mL tube with 4 mL of BPEL medium supplemented with 10% FBS. These incubation and dissociation steps were repeated five more times until the cardiac MTs were completely dissociated (5–7 times in total) for 10 min each time. The single-cell suspension was filtered using a 100 μm filter (CellTrics) and stained with anti-CD31-FITC antibody (1:50, Miltenyi) for 1h at 4°C. Finally, the cells were resuspended in BPEL supplemented with 0.5 mM EDTA (pH 8.0) and sorted for CD31^+^ cells using FACSAria III (BD-Biosciences).

#### Quantitative real-time PCR (qPCR)

Total RNA was extracted using the NucleoSpin RNA kit according to the manufacturer’s protocol. cDNA was synthesized using an iScript-cDNA Synthesis kit (Bio-Rad, Hercules, CA, USA). iTaq Universal SYBR Green Supermixes (Bio-Rad) and Bio-Rad CFX384 real-time system were used for the PCR reaction and detection. Relative gene expression was determined according to the standard ΔCT calculation and normalized to the housekeeping gene RPL37A. The primers used are shown in detail in Table S15.

#### Bulk RNA sequencing and analysis

Bulk RNAseq of passage two CMECs (CMECs P2) and human fetal heart ECs at gestation age Week(W)12.5, W15 and W21 were performed in our previous study^21^ and obtained from GEO accession number GSE116464.

Bulk RNAseq of day 6 and 8 of CMEC and PMEC differentiation were performed at BGI (Shenzhen, China) using the Illumina Hiseq4000 (100bp paired end reads). Raw data were processed using the LUMC BIOPET Gentrap pipeline (https://github.com/biopet/biopet), which comprises FASTQ preprocessing, alignment and read quantification. Sickle (v1.2) was used to trim low-quality read ends (https://github.com/najoshi/sickle). Cutadapt (v1.1) was used for adapter clipping,^56^ reads were aligned to the human reference genome GRCh38 using GSNAP (gmap-2014-12-23)^57^^,^^58^ and gene read quantification with htseq-count (v0.6.1p1) against the Ensembl v87 annotation.^59^ Gene length and GC content bias were normalized using the R package cqn (v1.28.1).^60^

Bulk RNAseq of ECs sorted on day 6 and 27 of CMEC differentiation, and day 27 MT_cmec were performed using VASA-seq. A 96-well plate (Greiner Bio-One, 652250) was filled with 50 μL mineral oil (Merck, M5904) and 1 μL CEL-seq2 primer^61^ per well with a concentration of 7.5 ng/μL. RNA samples were diluted to 20 ng/μL in nuclease free water. Of the dilution, 1 μL from each sample was added to different wells of the barcoded 96-well plate. The plates were sealed (Greiner Bio-One, 676090) and spun down at 500 × g for 2 min at 4°C. Next, library preparation for bulk samples was done as previously described.^62^ In short, RNA fragmentation was performed by adding 2 μL of fragmentation mixture per sample consisting of 0.68× First-strand buffer (Invitrogen, Y02321) and the rest, ddH2O. Fragmentation was carried out using a Thermal Cycler (Bio-Rad, T100) set at 85°C for 3 min with the lid at 105°C. Next, 2 μL of end repair and poly(A)-tailing mix consisting of 0.12× First-strand buffer (Invitrogen, Y02321), 20 mM DTT(Invitrogen, y00147), 7.5 μM ATP (NEB, P0756S), 0.15 U of E. coli Poly(A) Polymerase (NEB, M0267L), 2 U of T4 PNK (NEB, M0201L) and 8 U of RNaseOUT (Invitrogen, 10777019) was added to each well. Repair and tailing were done at 37°C in a Thermal Cycler for 1 h with the lid at 70°C. For reverse transcription, 2 μL of a mix consisting of 2 mM (each) dNTP (Solis Biodyne, 02-21-00400) and 32 U of SuperScript III (Invitrogen, 18080085) was added to each sample. Reverse transcription was done using a Thermal Cycler set at 50°C for 1 h with the lid at 70°C. The plates were snap-chilled on ice. Next, 11 μL of second strand mix containing 0.23× Second-Strand Buffer (Invitrogen, 10812014), 0.23 mM (each) dNTP, 3.5 U of E. coli DNA Polymerase I (Invitrogen, 18010025) and 0.2 U of RNaseH (Invitrogen, 18021071). Second strand synthesis was done at 16°C for 2 h, followed by 85°C for 20 min with the lid of the Thermo Cycler set to 105°C. After each aforementioned step, plates were spun down at 500xg for 2 min at 4°C. The samples were pooled together and the mineral oil was removed. Bead clean-up was performed by adding 1× volumetric ratio of diluted RNAClean XP beads (Beckman Coulter, A63987). The beads were diluted 1:2 in bead binding buffer (20% PEG8000 (Promega, V3011) and 2.5M NaCl). RNA was eluted in 6.4 μL of nuclease-free water. Next, 9.6 μL of IVT mix containing 12 mM MegaScript T7 buffer, T7 enzyme, ATP, CTP, GTP and UTP (Invitrogen, AM1334) was added to the pooled sample. IVT was done at 37°C for 14 h with the lid set to 70°C. Following IVT, 6 μL of ExoSAP-IT (Invitrogen, 75001.1.ML) was added and the sample was incubated at 37°C for 15 min with the lid set to 70°C. Concentration of aRNA was measured using a Qubit (Invitrogen, Q32853) and adjusted to 100 ng/μL. The rRNA depletion, adaptor ligation, cDNA synthesis, RNA degradation, PCR amplification and final clean-up was used as previously described,^62^ with a few modifications. The primers used are shown in detail in Table S16. Paired-end sequencing with a read length of 150 bp was performed on the Illumina NovaSeq sequencing platform. The first 8 bases of read 2 contain the unique molecular identifier, and the next 8 bases contain the sample barcode (specific to each well), while read 1 contains the transcript information. Reads 1 with a valid sample barcode were selected and trimmed using TrimGalore (v.0.6.6) with default parameters. In addition, homopolymers at the end of the read were also trimmed out. Next, in silico ribosomal depletion was performed as described in [2]. Resulting reads were mapped using STAR (v2.7.7a) with default parameters using the human GRCh38 genome (Ensembl 102). Deduplicated reads were removed using fumi-tools (v0.17.0). Count tables were generated using featureCounts (from subread-2.0.1) with the following options: -M -O --fraction -s 1 -t exon -g gene_name. We used the human custom made gtf file from^63^ that integrates different biotypes.

Differentially expressed genes were identified using generalized linear models as implemented in edgeR (3.24.3).^64^ *p*-values were adjusted using the Benjamini-Hochberg procedure and FDR ≤0.05 was considered significant. Analyses were performed using R (version 3.5.2). PCA plot was generated with the built-in R functions prcomp using transposed normalized RPKM matrix. Correlation among samples was calculated using cor function with spearman method and the correlation heatmap was generated with aheatmap function (NMF package). Gene ontology enrichment was performed using compareCluster function of clusterProfiler package (v3.10.1)^65^ and q ≤ 0.05 was considered significant.

#### Single-cell RNA sequencing and analysis

##### Library preparation and sequencing

Library preparation was performed as previously described.^21^ Briefly, single cells were loaded into the 10× Chromium Controller for library construction using the Single-Cell 3′ Library Kit, Version 2 Chemistry (10× Genomics, Pleasanton, CA, USA) according to the manufacturer’s protocol. Next, indexed cDNA libraries were sequenced on the HiSeq4000 platform. Single-cell expression was quantified using unique molecular identifiers (UMIs) by 10× Genomics’ “Cell Ranger” software.

The mean reads per cell for all eight datasets: CMEC (R1): 28,499; CMEC (R2): 29,388; PMEC (R1): 31,860; PMEC (R2): 38,415; CM_MT (R1): 39,319; CM_MT (R2): 29,741; PM_MT (R1): 36,726; PM_MT (R2): 26,421.

##### Single-cell RNAseq data pruning and normalization

For data pruning and normalization, the two replicates of each of the 4 conditions (CMEC, PMEC, CM_MT and PM_MT) were combined without batch correction. Then, cells with a low number of genes per cell (1200 [CMEC], 1200 [PMEC], 900 [CM_MT], 750 [PM_MT], see Figures S2A and S2B) were removed. Genes expressed in less than 2 of the remaining cells with a count of at most 1 were excluded from further analysis. Each combined dataset was normalized using the R package scran (V 1.14.6).^66^ Highly variable genes (HVGs) were calculated (improvedCV2 from the scran package) for each replicate of the combined datasets after excluding ribosomal genes [Ribosomal Protein Gene Database], stress markers^67^ and mitochondrial genes. For downstream analysis the top 5% HVGs were used after excluding proliferation^68^ and cell cycle^69^ related genes.

##### Cell cycle analysis and batch correction

For each combined dataset, cell cycle analysis was performed with the scran package using the cyclone function^70^ on normalized counts (Figure S2H). Cells with a G2/M score higher than 0.2 were considered to be in G2/M phase. Otherwise, they were classified as G1/S. Using this binary classifier as predictor, we regressed out cell cycle effects with the R package limma (V 3.42.2)^71^ applied to log-transformed normalized counts. Then, for each combined dataset, the two replicates were batch corrected with fast mutual nearest neighbors correction method (MNN)^72^ on the cell cycle corrected counts, using the 30 first principal components and 20 nearest-neighbors (Figure S2C).

##### Clustering

For each combined dataset, batch-corrected counts were standardized per gene and then used to create a shared nearest neighbor (SNN) graph with the *scran* R package (d = 30, k = 2). Louvain clustering was applied to the SNN graph using the *igraph* python package (V 0.7.1) with these resolution parameters: 0.4 [CMEC], 0.4 [CM_MT], 0.3 [PMEC], 0.1 [PM_MT]. For the CMEC dataset, this resulted in 5 clusters (Figure S2D). Two of these 5 clusters were excluded from further analysis based on the expression of pluripotency markers (Figure S2E). For the PMEC dataset, this resulted in 3 clusters (Figure 2F). For CM_MT and PM_MT, clustering resulted in 4 clusters (Figures S2F and S2G), where one cluster was excluded from further analysis, because it was mainly present in one of the two replicates. Additionally, the attempt to map this cluster to *in vivo* data, resulted in mostly unassigned cell types (plot not shown). For PMEC, clustering resulted in 3 clusters.

##### Dimensionality reduction and pseudotime

Dimensionality reduction was performed using the python *scanpy* pipeline (V 1.4.6). For both datasets, CMEC and PMEC, a 20 nearest-neighbors (knn, k = 20) graph was created from diffusion components of the batch corrected datasets. Diffusion components are the eigenvectors of the diffusion operator which is calculated from Euclidean distances and a Gaussian kernel. The aim is to find a lower dimensional embedding which considers the cellular progression. Both graphs were projected into two dimensions with the default force-directed graph layout and starting positions obtained from the partition-based graph abstraction (PAGA) output.^73^ PAGA estimates connectivities between partitions and performs an improved version of diffusion pseudotime. Diffusion pseudotime^72^^,^^73^ was calculated on these graphs with root cells selected based on the graph layout from the “Cardiac Mesoderm” cluster in CMEC, and the “Paraxial Mesoderm” cluster in PMEC.

For CM_MT and PM_MT, the knn graphs (k = 50 for PM_MT, k = 100 for CM_MT) were created from the first 30 principal components of the batch corrected datasets. These graphs were projected into two dimensions with the default force-directed graph layout and starting positions from the PAGA output.

##### *In vivo* data analysis and mapping

The *in vivo* dataset, downloaded from https://www.spatialresearch.org/resources-published-datasets/doi-10-1016-j-cell-2019-11-025/, contains a 6.5 PCW human embryonic cardiac tissue sample. The clusters and cluster annotations were obtained from the original publication.^34^ The dataset was normalized with the scran R package and HVGs were calculated as described in section “single-cell RNAseq data pruning and normalization”. Dimensionality reduction was performed with the R package *umap* (V 0.2.5.0) using 20 nearest-neighbors, min_dist = 0.7 and Euclidean distance.

##### Differential expression analysis

All differential expression tests were performed with edgeR (V 3.28.1)^64^ using a negative binomial regression and raw counts. The predictors in the regression were: cluster and replicate (both discrete variables), as well as the total number of counts per cell.

For marker gene analysis (Figures S3A and S3C), *p*-values were obtained for a contrast between the cluster of interest and all other clusters using regression coefficients averaged over the replicates. For tests between different datasets (Figure 3C), the corresponding endothelial cell cluster was extracted from each dataset. Then, a contrast between MT and day 6 was calculated by averaging over the predictors of both replicates. For the *in vivo* test (Figure S4B), intra-myocardial EC and endocardium clusters were extracted from the dataset to calculate the contrast between them. *p*-values were adjusted for multiple hypothesis testing with the Benjamini-Hochberg method.

##### Comparison to the *in vivo* dataset

MT_cmec and MT_pmec datasets were mapped on the *in vivo* dataset using the MNN method (d = 30 principal components, k = 100 nearest neighbors). First, *in vitro* replicates were mapped to each other, then the *in vivo* data were mapped on the combined *in vitro* data, using normalized, log-transformed counts and the 10% top HVGs of the *in vivo* dataset. Dimensionality reduction was performed with the R package *umap* using 100 nearest-neighbors, min_dist = 0.3 and Euclidean distance.

K-nearest-neighbor (KNN) assignment was performed in the batch corrected, principal component space (30 PCs). The 100 nearest-neighbors in the *in vivo* dataset based on Euclidean distances were calculated for each *in vitro* cell. The *in vitro* cell was ascribed the cell type most abundant among the 100 *in vivo* neighbors. Each such assignment received a confidence score, which is the number of *in vivo* neighbors with that cell type divided by the number of all nearest neighbors (=100). A cell was not ascribed a cell type if either the average distance to its nearest neighbor exceeded a certain threshold (determined by the long tail of the histogram of average distances: 0.35), or the assignment had a confidence score less than 0.5. In addition, clusters containing less than 10 cells were not ascribed a cell type.

For the Jaccard similarity measure, marker genes of each differential expression test were selected with adjusted *p*-value <0.05. The remaining genes were ranked by log2 fold change, and the first 478 genes were selected for analysis. Then, the Jaccard distances were calculated between the marker genes of intra-myocardial endothelial cells and each of the other gene sets.

For principal component analysis (Figure 4H), human fetal bulk samples and *in vitro* bulk samples^21^ were combined with the single cell datasets. For each single-cell dataset, the endothelial cells were extracted and the sum per gene over all cells was calculated. Then, bulk and single cell samples were log-transformed and combined into one dataset. Principal component analysis was applied on the gene-wise standardized dataset, using marker genes of the intra-myocardial endothelial cells from the *in vivo* dataset.

### Quantification and statistical analysis

Statistical analysis was conducted with GraphPad Prism 7 software (San Diego, CA, USA). Data are represented as mean ± SD. Methods to examine differences between groups were described in Figure Legends. Statistical significance was set at ∗*p* < 0.05, ∗∗*p* < 0.01, ∗∗∗*p* < 0.001.

## Supplemental information

Document S1. Figures S1–S6 and Tables S15 and S16

Table S1. DEGs between bulk_CMEC_d6 and bulk_PMEC_d6, related to Figure 1E (A) Genes expressed higher in bulk_CMEC_d6 than bulk_PMEC_d6. (B) Genes expressed higher in bulk_PMEC_d6 than bulk_CMEC_d6.

Table S2. GOs enriched in DEGs between bulk_CMEC_d6 and bulk_PMEC_d6, related to Figure 1F

Table S3. Markers each cluster of CMEC differentiation day 6 by scRNAseq, related to Figure 2C and S3A

Table S4. Markers each cluster of PMEC differentiation day 6 by scRNAseq, related to Figure 2F and S3C

Table S5. Markers of each cluster in MT_cmec and MT_pmec by scRNAseq, related to Figure 3B. (A) Markers of MT_cmec. (B) Markers of MT_pmec

Table S6. DEGs between intramyocardial and endocardial ECs of human heart, related to Figure S4B and Figure 3

Table S7. DEGs between ECs on day 6 and in MTs by scRNAseq, related to Figure 3D-E and S5A-B (A) DEGs between sc_PMEC_mt and sc_PMEC_d6. (B) DEGs between sc_CMEC_mt and sc_CMEC_d6.

Table S8. DEGs between sc_CMEC_mt and sc_PMEC_mt, related to Figure 3F and S5C

Table S9. DEGs between bulk_CMEC_d6 and bulk CMEC_d27, related to Figure S6B and Figure 4 (A) Genes expressed higher in bulk CMEC_d27 than bulk_CMEC_d6. (B) Genes expressed higher in bulk_CMEC_d6 than bulk CMEC_d27.

Table S10. DEGs between bulk_CMEC_d27 and bulk_CMEC_mt, related to Figure S6B and Figure 4 (A) Genes expressed higher in bulk_CMEC_mt than bulk_CMEC_d27. (B) Genes expressed higher in bulk_CMEC_d27 than bulk bulk_CMEC_mt.

Table S11. DEGs between bulk_CMEC_d6 and bulk_CMEC_mt, related to Figure S6B and Figure 4 (A) Genes expressed higher in bulk_CMEC_mt than bulk_CMEC_d6. (B) Genes expressed higher in bulk_CMEC_d6 than bulk bulk_CMEC_mt.

Table S12. DEGs between sc_CMEC_d6 and sc_PMEC_d6, related to Figure 4G

Table S13. GOs for gene lists shown in Figure4G, related to Figure 4G

Table S14. Complete list of GOs for Figure S6C, related to Figure S6C (A) GOs that enriched in DEGs that expressed higher in bulk_CMEC_d27 than bulk_CMEC_d6. (B) GOs that enriched in DEGs that expressed higher in bulk_CMEC_mt than bulk_CMEC_d6. (C) GOs that enriched in DEGs that expressed higher in bulk_CMEC_mt than bulk_CMEC_d27.
