# Supplementary material for: Tissue microenvironment dictates the state of human iPSC-derived endothelial cells of distinct developmental origin in 3D cardiac microtissues
Source: iScience. 2025 Sep 22;28(10):113611. doi: 10.1016/j.isci.2025.113611 (PMC12546991; doi:10.1016/j.isci.2025.113611)
Supplement: Document S1. Figures S1–S6 and Tables S15 and S16 [file mmc1.pdf]

iScience, Volume *28*

Supplemental information

Tissue microenvironment dictates the state of human iPSC-derived endothelial cells of distinct developmental origin in 3D cardiac microtissues

Xu Cao, Maria Mircea, Sara Cascione, Atoosa Amel, Theano Tsikari, Francijna E. van den Hil, Hailiang Mei, Katrin Neumann, Anna Alemany, Konstantinos Anastassiadis, Christine L. Mummery, Stefan Semrau, and Valeria V. Orlova

Figure S1.

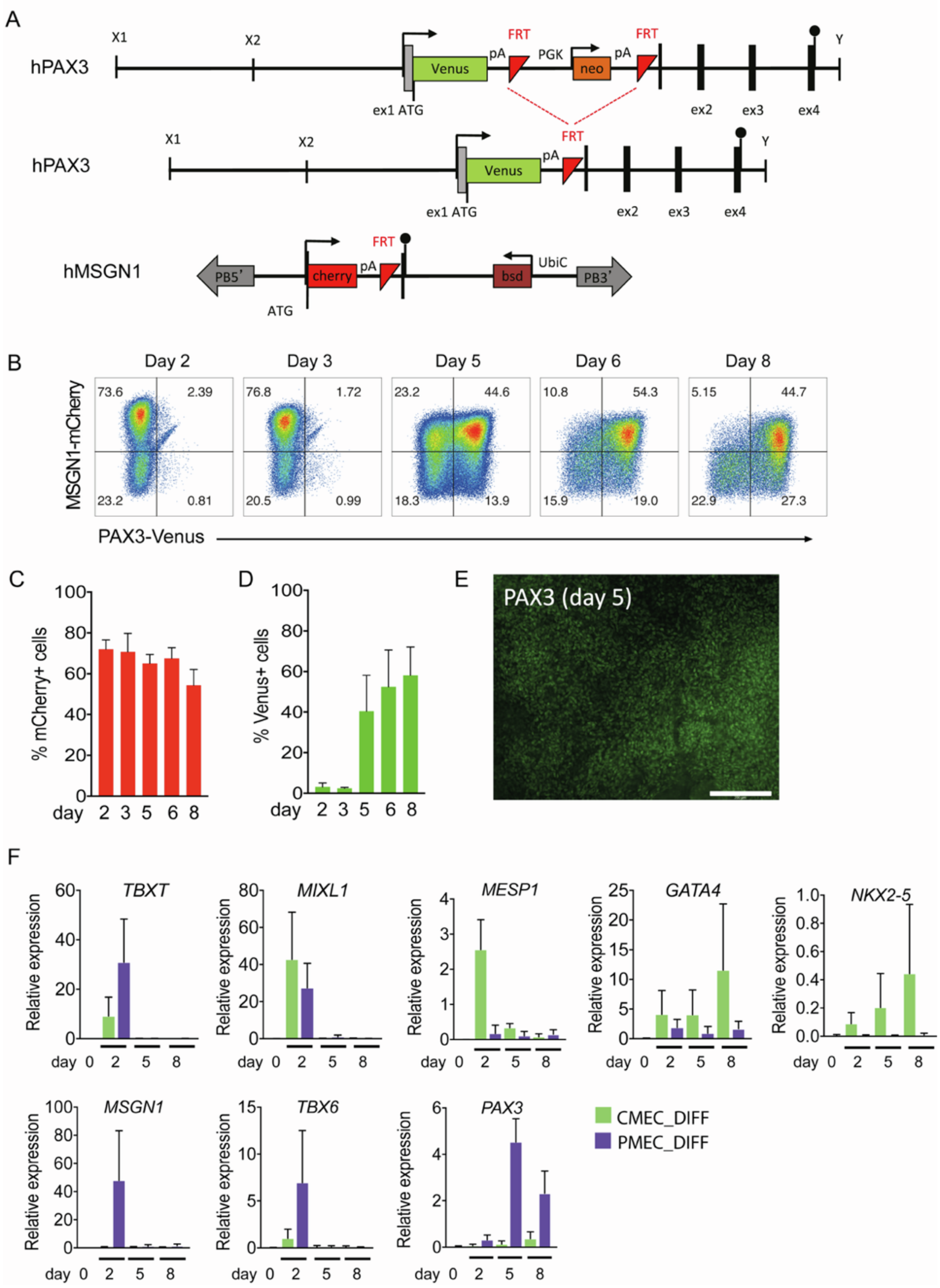

Figure S1. Characterization of PMEC differentiation using MSGN1^{mCherry}PAX3^{Venus} dual reporter line, Related to Figure 1.

(**A**) Targeting constructs used to generate PAX3-Venus and MSGN1-mCherry hiPSC reporter line. (**B**) Flow cytometry analysis of PAX3^{Venus} and MSGN1^{mCherry} expression on day 2, 3, 5, 6 and 8 of PMEC differentiation. (**C-D**) Quantification of mCherry+ (**C**) and Venus+ (**D**) cells in the total population by flow cytometry on day 2, 3, 5, 6 and 8. (**E**) Representative immunofluorescence staining of PAX3 on day 5 of PMEC differentiation. Scale bar: 200 μm. (**F**) Quantification of *TBXT, MIXL1, MESP1, GATA4, NKX2-5, MSGN1, TBX6* and *PAX3* expression by qPCR on day 0, 2, 5 and 8 of CMEC (green) and PMEC (purple) differentiation. For panels C, D and F, data were collected from three independent experiments. Data are shown as mean ± SD.

Figure S2.

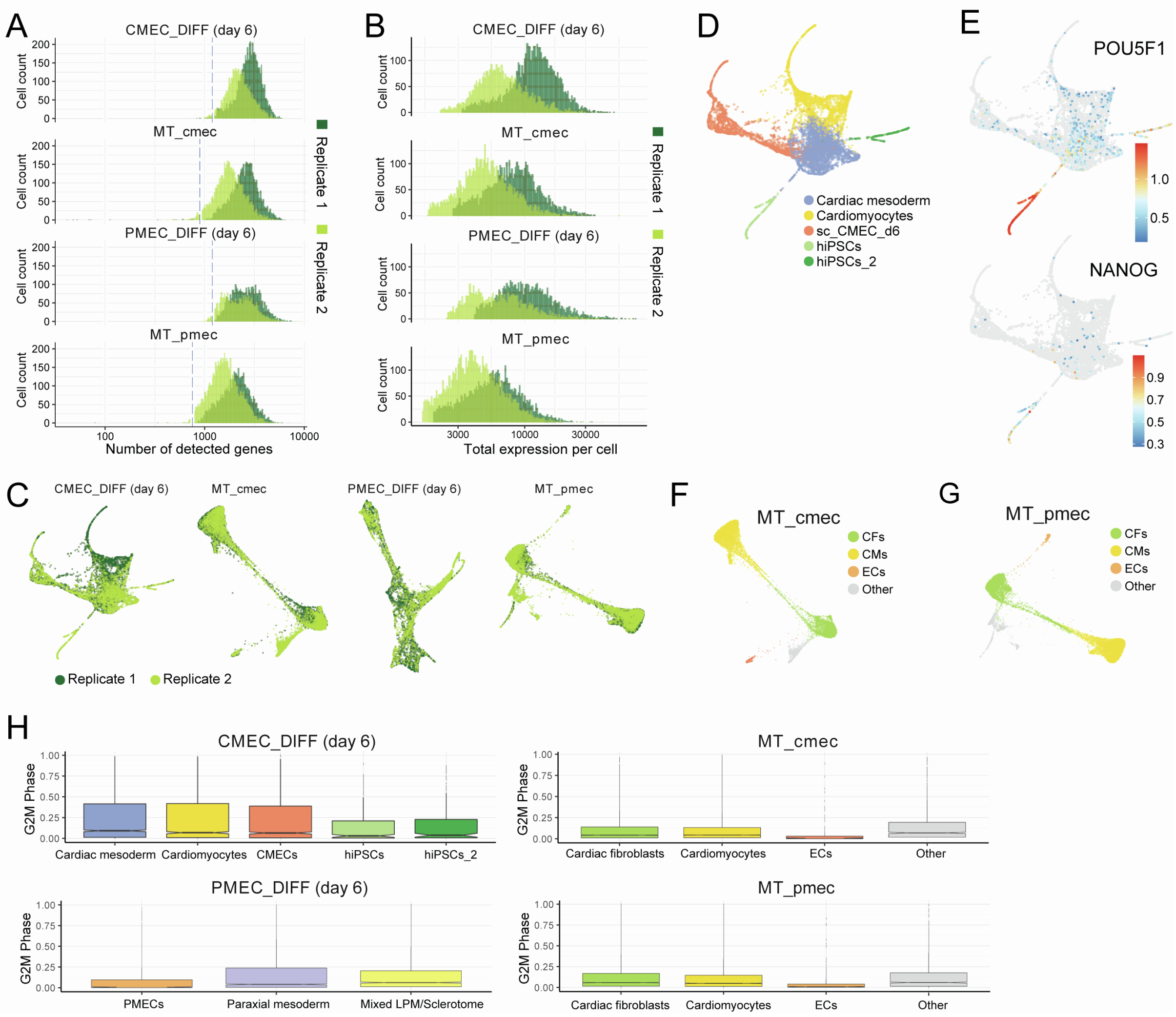

Figure S2. Quality control of scRNA-seq datasets, Related to Figure 2.
(**A-B**) Distribution of the number of detected genes (**A**) and total expression (**B**) in each cell of the scRNA-seq datasets. The dotted blue lines indicate quality control thresholds. Two different batches are labelled with different colors. (**C**) Two different batches of cells collected for each scRNA-seq dataset were visualized with PAGA. (**D**) scRNA-seq data of CMECs on day 6 is visualized using PAGA. Five cell clusters were identified and labelled with different colors. (**E**) Expression of pluripotency genes *POU5F1* and *NANOG* in the CMEC dataset on day 6 is shown in PAGA plot. Color represents log transformed expression. (**F-G**) scRNA-seq data of MT_cmec (**F**) and MT_pmec (**G**) were visualized using PAGA. Four cell clusters were identified. Clusters labelled with “Other” were excluded from downstream analysis. (**H**) Boxplot of G2M phase-score in individual clusters of each dataset.

Figure S3.

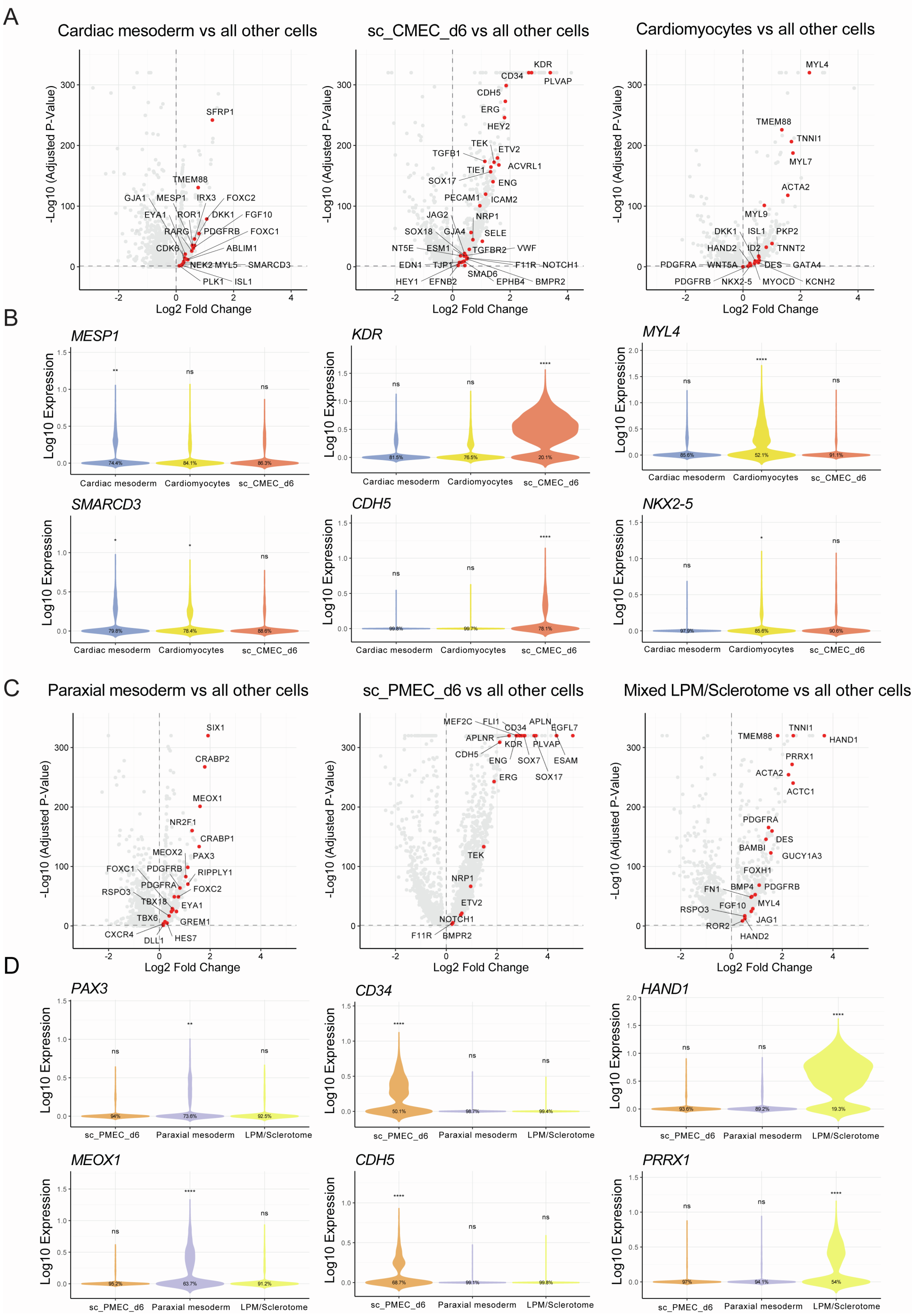

Figure S3. scRNA-seq analysis of CMEC and PMEC datasets on day 6, Related to Figure 2.
(**A**) Volcano plots showing fold changes and p-values of differential expression tests between individual CMEC clusters and all other cells. Representative significantly up-regulated genes ($p_{adjusted} < 0.05$ & fold-change > 1.2) are labelled in red. (**B**) *MESP1*, *SMARCD3*, *KDR*, *CDH5*, *MYL4* and *NKX2-5* expression (log transformed) in three clusters of the CMEC dataset on day 6. (**C**) Volcano plots showing fold-changes and p-values of differential expression tests between individual PMEC clusters and all other cells. Representative significantly up-regulated genes ($p_{adjusted}<0.05$ & fold change > 1.2) are labelled in red. (**D**) *PAX3*, *MEOX1*, *CD34*, *CDH5*, *HAND1* and *PRRX1* expression (log transformed) in three clusters of PMEC dataset on day 6.

Figure S4.

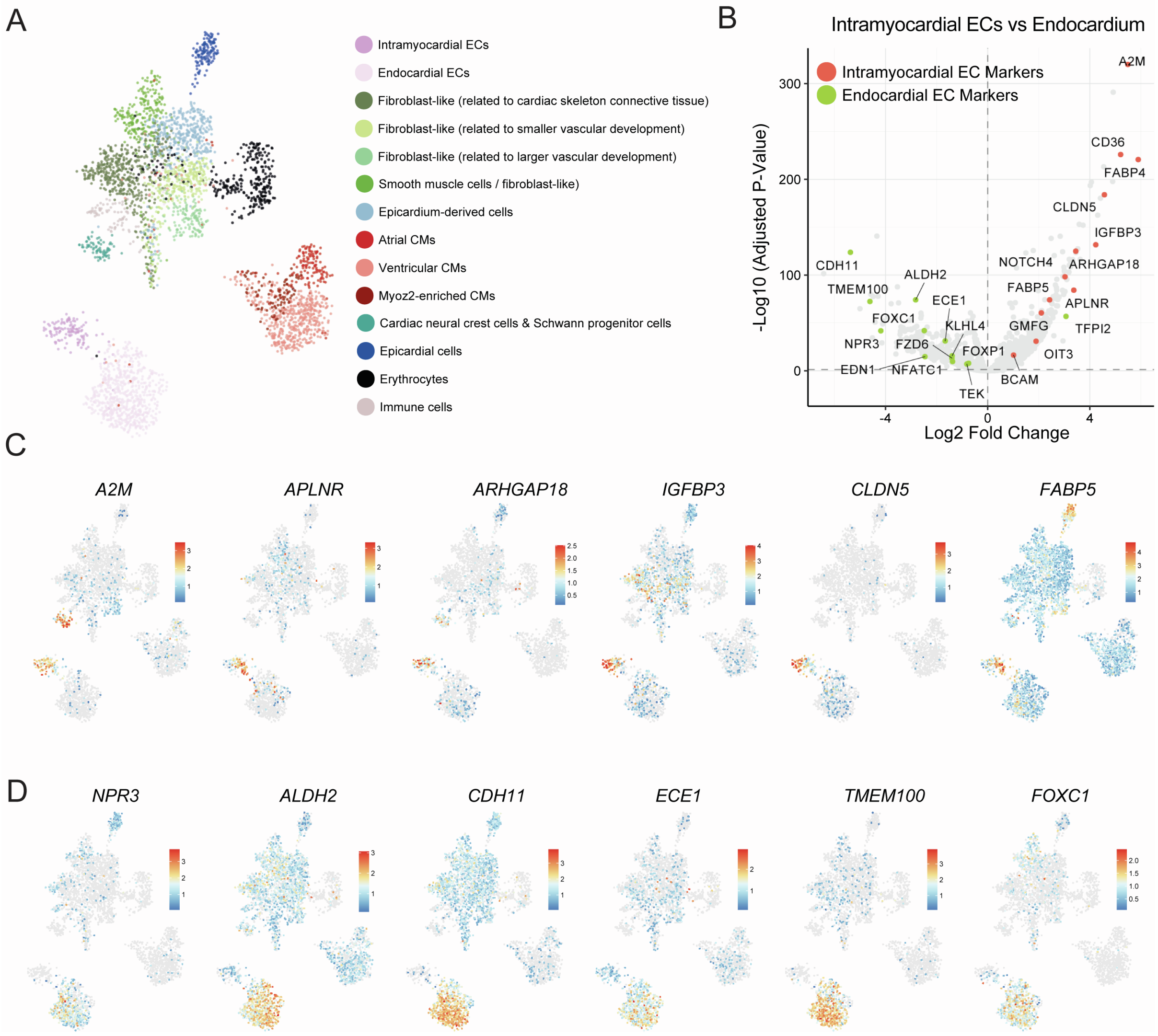

Figure S4. Re-analysis of a published scRNA-seq dataset to identify organ specific signatures of human fetal heart ECs, Related to Figure 3.
(**A**) Low-dimensional representation (UMAP) of scRNA-seq of the human fetal heart (Asp et al., 2019). 14 cell clusters were identified and named based on the original publication, except for two EC clusters: intramyocardial ECs and endocardium. (**B**) Volcano plot showing fold changes and p-values for differential expression tests between intramyocardial ECs and endocardium in the data set shown in (**A**). Representative differentially expressed genes ($p_{adjusted}<0.05$) that are known as intramyocardial and endocardial markers are labelled in red and green respectively. (**C-D**) Low-dimensional representation (UMAP) of scRNA-seq of the human fetal heart (Asp et al., 2019). Log-transformed expression of representative intramyocardial EC markers (**C**) and endocardium markers (**D**) is indicated by color.

Figure S5.

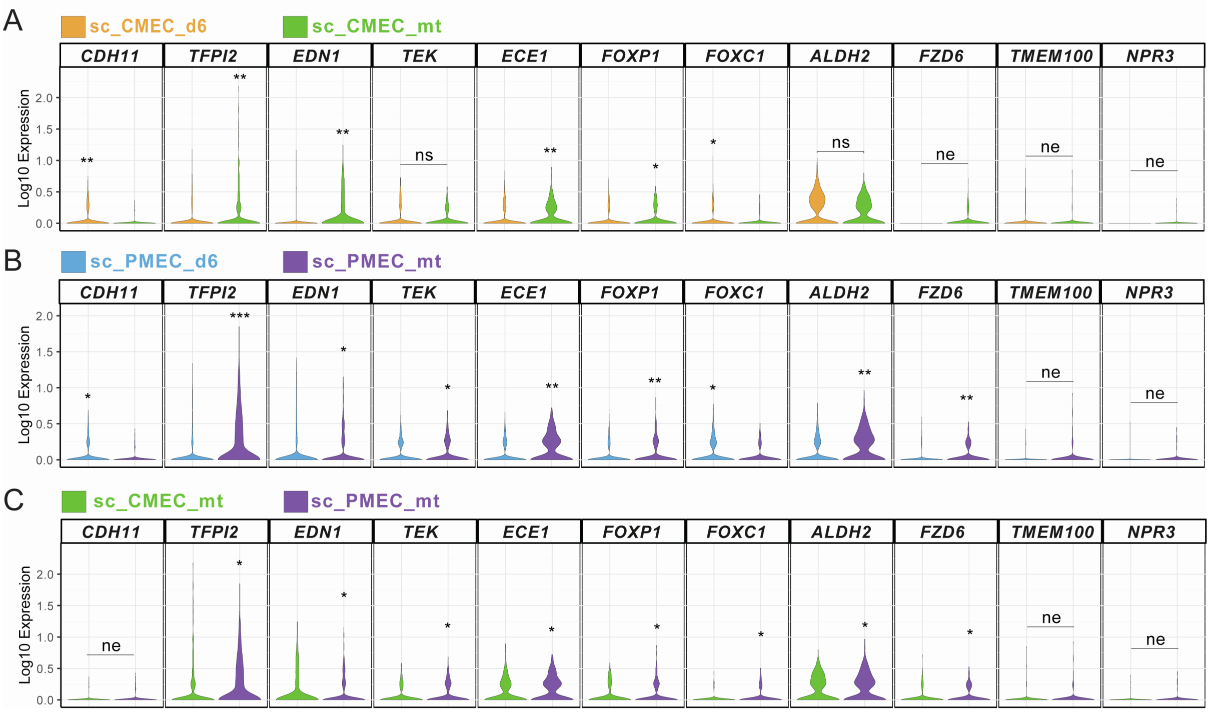

Figure S5. Comparison of organ-specific signatures of hiPSC-ECs on day 6 with ECs in MTs, Related to Figure 3.

(**A-C**) Differential expression test between clusters sc_CMEC_d6 and sc_CMEC_mt (**A**), sc_PMEC_d6 and sc_PMEC_mt (**B**), sc_CMEC_mt and sc_PMEC_mt (**C**) for representative endocardial EC markers. ns: p>0.05; * p<=0.05; ** p<= 1e-10; *** p<= 1e-100; **** p<= 1e-200. Clusters with higher expression value were indicated with stars. ne: not expressed (0 counts) in >85% of cells in both groups.

Figure S6.

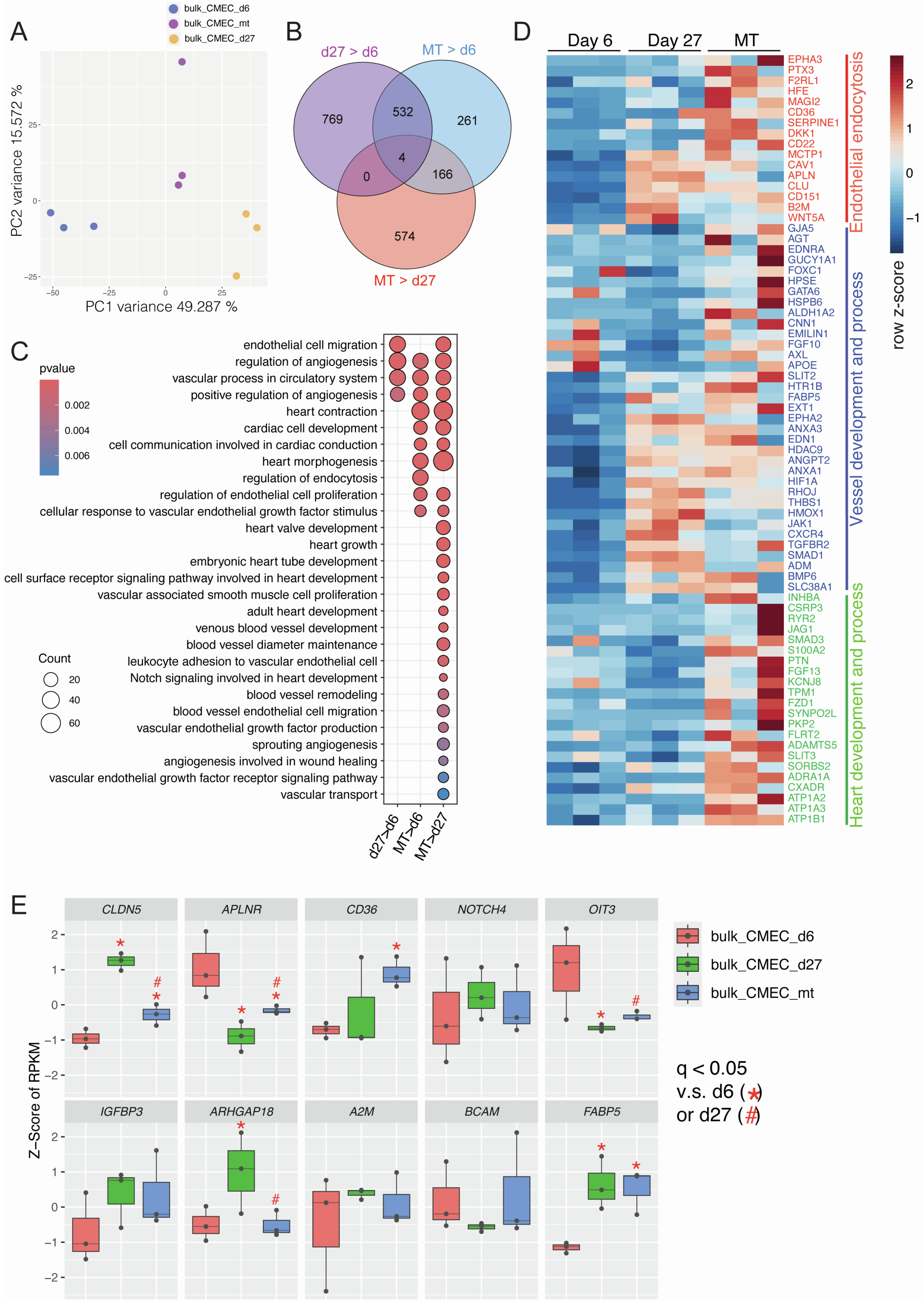

Figure S6. Transcriptomic analysis of age-matched CMECs in 2D monoculture and 3D MTs, related to Figure 4.
(**A**) PCA plot of CMECs on day 6 (bulk_CMEC_d6), day 27 (bulk_CMEC_d27) and CMECs sorted from MTs on day 27 (bulk_CMEC_mt). Data were collected from three independent experiments. (**B**) Number of DEGs between selected groups as indicated. q < 0.05. (**C**) Representative GO enrichments for these three lists of DEGs as shown in (**B**). (**D**) Expression pattern of selected genes in GOs identified in (**C**). (**E**) Expression of intramyocardial EC markers among different groups of CMECs. * q<0.05 compared bulk_CMEC_d6. # q<0.05 compared to bulk_CMEC_d27.

Table S15. Primer sequences for RT-qPCR, related to STAR Methods

Gene symbol	Sense	Anti-sense
TBXT	TATGAGCCTCGAATCCACATAGT	CCTCGTTCTGATAAGCAGTCAC
MIXL1	GGCGTCAGAGTGGGAAATCC	GGCAGGCAGTTCACATCTACC
MESP1	AGCTGCACCCGAGCCGCGC	ATCCAGGTCTCCAACAGAGCCA
GATA4	CGACACCCCAATCTCGATATG	GTTGCACAGATAGTGACCCGT
NKX2-5	TCTATCCACGTGCCTACAGC	GTTGTCCGCCTCTGTCTTCT
MSGN1	AACCTGCGCGAGACTTTCC	GTCTGTGAGTTCCCCGATGTA
TBX6	ATCTCCGTGACAGCCTACCA	CCGCAGTTTCCTCTTCACAC
PAX3	GACTTGGAGAGGAAGGAGGC	CTTCATCTGATTGGGGTGCT

Table S16. Primer sequences for VASAseq, related to STAR Methods

Sample name	condition	day	origin	line	VASA barcode	VASA_primer
sample_01	D6_CMEC	day 6	CMEC	SLC098_99	ATGCCTCA 241	GCC GGT AAT ACG ACT CAC TAT AGG CCT TGG CAC CCG AGA ATT CCA ATG CCT CAA TGC CTC ATT TTT TTT TTT TTT TTT TTT TTT TTV N
sample_02	D6_PMEC	day 6	PMEC	SLC098_99	TCCGAACA 242	GCC GGT AAT ACG ACT CAC TAT AGG CCT TGG CAC CCG AGA ATT CCA TCC GAA CAT CCG AAC ATT TTT TTT TTT TTT TTT TTT TTT TTV N
sample_03	D27_CMEC	day 27	CMEC	SLC098_99	GCATCAGT 251	GCC GGT AAT ACG ACT CAC TAT AGG CCT TGG CAC CCG AGA ATT CCA GCA TCA GTG CAT CAG TTT TTT TTT TTT TTT TTT TTT TTT TTV N
sample_04	D27_PMEC	day 27	PMEC	SLC098_99	CAATACGC 252	GCC GGT AAT ACG ACT CAC TAT AGG CCT TGG CAC CCG AGA ATT CCA CAA TAC GCC AAT ACG CTT TTT TTT TTT TTT TTT TTT TTT TTV N
sample_05	MT_CMEC	MT on day 27	CMEC	SLC098_99	GAGGGTAG 059	GCC GGT AAT ACG ACT CAC TAT AGG CCT TGG CAC CCG AGA ATT CCA GAG GGT AGG AGG GTA GTT TTT TTT TTT TTT TTT TTT TTT TTV N
sample_06	MT_PMEC	MT on day 27	PMEC	SLC098_99	CGGGTGAA 060	GCC GGT AAT ACG ACT CAC TAT AGG CCT TGG CAC CCG AGA ATT CCA CGG GTG AAC GGG TGA ATT TTT TTT TTT TTT TTT TTT TTT TTV N
sample_07	D6_CMEC	day 6	CMEC	SLC101_102	GTGTTCAG 289	GCC GGT AAT ACG ACT CAC TAT AGG CCT TGG CAC CCG AGA ATT CCA GTG TTC AGG

						TGT TCA GTT TTT TTT TTT TTT TTT TTT TTT TTV N
sample_ 08	D6_PMEC	day 6	PMEC	SLC1 01_10 2	TCTCGT CT 290	GCC GGT AAT ACG ACT CAC TAT AGG CCT TGG CAC CCG AGA ATT CCA TCT CGT CTT CTC GTC TTT TTT TTT TTT TTT TTT TTT TTT TTV N
sample_ 09	D27_CME C	day 27	CMEC	SLC1 01_10 2	AGTTCT CG 299	GCC GGT AAT ACG ACT CAC TAT AGG CCT TGG CAC CCG AGA ATT CCA AGT TCT CGA GTT CTC GTT TTT TTT TTT TTT TTT TTT TTT TTV N
sample_ 10	D27_PME C	day 27	PMEC	SLC1 01_10 2	ACCAAG GA 300	GCC GGT AAT ACG ACT CAC TAT AGG CCT TGG CAC CCG AGA ATT CCA ACC AAG GAA CCA AGG ATT TTT TTT TTT TTT TTT TTT TTT TTV N
sample_ 11	MT_CME C	MT on day 27	CMEC	SLC1 01_10 2	TCTGCC TA 107	GCC GGT AAT ACG ACT CAC TAT AGG CCT TGG CAC CCG AGA ATT CCA TCT GCC TAT CTG CCT ATT TTT TTT TTT TTT TTT TTT TTT TTV N
sample_ 12	MT_PME C	MT on day 27	PMEC	SLC1 01_10 2	CTGGCT AA 108	GCC GGT AAT ACG ACT CAC TAT AGG CCT TGG CAC CCG AGA ATT CCA CTG GCT AAC TGG CTA ATT TTT TTT TTT TTT TTT TTT TTT TTV N
sample_ 13	D6_CME C	day 6	CMEC	SLC1 03_10 4	GTTATG CC 337	GCC GGT AAT ACG ACT CAC TAT AGG CCT TGG CAC CCG AGA ATT CCA GTT ATG CCG TTA TGC CTT TTT TTT TTT TTT TTT TTT TTT TTV N
sample_ 14	D6_PMEC	day 6	PMEC	SLC1 03_10 4	CGAACT GT 338	GCC GGT AAT ACG ACT CAC TAT AGG CCT TGG CAC CCG AGA ATT CCA CGA ACT GTC GAA CTG TTT TTT TTT TTT TTT TTT TTT TTT TTV N
sample_ 15	D27_CME C	day 27	CMEC	SLC1 03_10 4	ACAAGG CA 347	GCC GGT AAT ACG ACT CAC TAT AGG CCT TGG CAC CCG AGA ATT CCA ACA AGG CAA CAA GGC ATT TTT TTT TTT TTT TTT TTT TTT TTV N
sample_ 16	D27_PME C	day 27	PMEC	SLC1 03_10 4	AGTTTC GG 348	GCC GGT AAT ACG ACT CAC TAT AGG CCT TGG CAC CCG AGA ATT CCA AGT TTC GGA GTT TCG GTT TTT TTT TTT TTT TTT TTT TTT TTV N
sample_ 17	MT_CME C	MT on day 27	CMEC	SLC1 03_10 4	TGGTTG TC 155	GCC GGT AAT ACG ACT CAC TAT AGG CCT TGG CAC CCG AGA ATT CCA TGG TTG TCT GGT TGT CTT TTT TTT TTT TTT TTT TTT TTT TTV N
sample_ 18	MT_PME C	MT on day 27	PMEC	SLC1 03_10 4	AGGCAT CT 156	GCC GGT AAT ACG ACT CAC TAT AGG CCT TGG CAC CCG AGA ATT CCA AGG CAT CTA GGC ATC TTT TTT TTT TTT TTT TTT TTT TTT TTV N
